# Sophoraflavanone G from Phit-Sanat (*Sophora Exigua* Craib) inhibits WT1 protein expression and induces cell cycle arrest and apoptosis in acute myeloid leukemia

**DOI:** 10.1186/s12906-025-05116-1

**Published:** 2025-10-08

**Authors:** Lapamas Rueankham, Lokadi Pierre Luhata, Pawaret Panyajai, Natsima Viriyaadhammaa, Sawitree Chiampanichayakul, Methee Rungrojsakul, Trinnakorn Katekunlaphan, Siriporn Okonogi, Pronngarm Dejkriengkraikul, Aroonchai Saiai, Toyonobu Usuki, Colleen Sweeney, Songyot Anuchapreeda

**Affiliations:** 1 Department of Medical Technology, Faculty of Associated Medical Sciences, Chiang Mai University, Chiang Mai, Thailand; 2 Department of Materials and Life Sciences, Faculty of Science and Technology, Sophia University, Tokyo, Japan; 3 Cancer Research Unit of Associated Medical Sciences (AMS CRU), Faculty of Associated Medical Sciences, Chiang Mai University, Chiang Mai, Thailand; 4 Center of Excellence in Pharmaceutical Nanotechnology, Faculty of Pharmacy, Chiang Mai University, Chiang Mai, Thailand; 5 Department of Traditional Chinese Medicine, Faculty of Science, Chandrakasem Rajabhat University, Bangkok, Thailand; 6 Department of Chemistry, Faculty of Science, Chandrakasem Rajabhat University, Bangkok, Thailand; 7 Department of Pharmaceutical Science, Faculty of Pharmacy, Chiang Mai University, Chiang Mai, Thailand; 8 Department of Biochemistry, Faculty of Medicine, Chiang Mai University, Chiang Mai, Thailand; 9 Department of Chemistry, Faculty of Science, Chiang Mai University, Chiang Mai, Thailand; 10 Department of Biochemistry and Molecular Medicine, UC Davis Comprehensive Cancer Center, UC Davis School of Medicine, Sacramento, CA USA

**Keywords:** Phit sanat, *Sophora exigua* Craib, Leukemia, WT1, Sophoraflavanone G

## Abstract

**Background:**

*Sophora Exigua *Craib, also known as Phit-Sanat in Thai, belongs to the Fabaceae family. The root of *S. exigua* has been used in Kheaw-Hom, a Thai traditional remedy, for fever treatment. Bioactive compounds from *S. exigua* have been reported to exhibit health-promoting effects, including anticancer activity. However, their anti-leukemic properties have not yet been elucidated.

**Methods:**

The study employed the MTT assay to evaluate the cytotoxic effects on leukemic cell lines (KG-1a and EoL-1) and PBMCs. Active compounds were purified using column chromatography and further characterized by NMR spectroscopy. Cell cycle distribution and apoptosis were assessed using appropriate kits and analyzed via flow cytometry. The expression of Wilms’ tumor 1 (WT1) protein was examined by Western blot analysis. Proteomic analysis was conducted using online software to investigate gene ontology (GO) and Kyoto Encyclopedia of Genes and Genomes (KEGG) pathway enrichment.

**Results:**

The ethyl acetate (EtOAc) crude fractional extract from *S. exigua* No. 010 (collected from Chaiyaphum province, Thailand) exhibited strong cytotoxicity in vitro toward both KG-1a and EoL-1 cells. Two active compounds, sophoraflavanone G (SG) and exiguaflavanone B (EGF-B), were isolated from EtOAc No. 010. EtOAc No. 010, SG, and EGF-B suppressed the proliferation of KG-1a and EoL-1 cells in a time- and dose-dependent manner by inducing G1 cell cycle arrest and apoptotic cell death. In this study, EtOAc No. 010, SG, and EGF-B were found to reduce WT1 expression in KG-1a and EoL-1 cells in a dose-dependent manner, with SG exhibiting greater activity than EGF-B. Additionally, KEGG pathway enrichment analysis of the differentially expressed proteins in KG-1a cells following SG treatment revealed significant enrichment in cell cycle regulation, apoptosis, and in the pathways associated with WT1protein expression, including AMPK, VEGF, and mTOR pathways.

**Conclusion:**

The SG isolated from *S. exigua*, exerts antiproliferative activity towards leukemic cells.

**Supplementary information:**

The online version contains supplementary material available at 10.1186/s12906-025-05116-1.

## Introduction

The *Sophora* genus belongs to the Fabaceae family and is widely found in Asia and Africa. The traditional medicinal use of *Sophora* species, such as *S. flavescens*, *S. tokinensis*, and *S. alopecuroides*, is well documented, with these plants being used to treat various diseases [1]. *S. exigua* Craib, known as Phit-Sanat in Thai, is a small shrub distributed in the upper northeastern region of Thailand and Cambodia. Its roots are well known for their used in Kheaw-Hom, a Thai traditional remedy for the treatment of exanthematous fever such as chickenpox, measles, and herpes zoster [2], as well as for promoting postpartum breast milk production in women. Previous studies have reported that *S. exigua* extracts exhibit antioxidant [3] and antimicrobial activity against *Pseudomonas aeruginosa*, *Staphylococcus epidermidis*, and *Candida albicans* [4]. In 2021, the antimalarial activity of the ethanolic extract of *S. exigua* roots was reported [5]. Compounds isolated from *S. exigua* were identified and included flavonols, flavones, chromones, and pterocarpans. The phenolic compounds in the root of *S. exigua* were characterized as exiguaflavanones A–M and a benzochromone, exiguachromone B [6–8]. One of the most important bioactive compounds isolated from members of the *Sophora* genus, including *S. leachina*, *S. exigua*, *S. moorcroftiana*, *S. pachycarpa*, and *S. flavescens*, is sophoraflavanone G (5,7,2’,4’-tetrahydroxy-8-lavandulylflavanone; SG). This compound exhibits outstanding pharmacological properties, demonstrates potent antibacterial activity against methicillin-resistant *Staphylococcus aureus* (MRSA), and reduces bacterial cell membrane fluidity [9, 10]. Moreover, SG has a highly lipophilic structure, which contributes to its strong affinity for the cell membrane and enhances its biological activities, including estrogenic, antibacterial, and anti-inflammatory effects [11]. Currently, cytotoxic chemotherapy is widely used in cancer treatment. However, these drugs affect both cancer and normal cells, leading to adverse effects such as hair loss, nausea, vomiting, diarrhea, etc [12]. The development of alternative therapies with low toxicity to normal cells and tissues is crucial for improving cancer treatment efficacy. Traditional medicine has long utilized herbs and medicinal plants for disease prevention and treatment. Certain bioactive compounds derived from medicinal plants exhibit anticancer properties by suppressing tumor cell proliferation, modulating the immune system and inhibiting angiogenesis. Some of these compounds are clinically used as cancer treatment. Moreover, several studies have indicated that bioactive compounds derived from medicinal plants exhibit various biological and pharmacological properties while being non-toxic to normal cells and approved for clinically treatment [13, 14]. Cytotoxicity in normal cells is always assessed to ensure safety and minimize side effects. If certain medicinal plant extracts or natural compounds exhibit cytotoxicity, a non-cytotoxic dose for normal cells can be selected. However, this non-toxic concentration should still be effective against targeted cancer cells. For example, *Cephalotaxus harringtonia* is well known in traditional Chinese medicine. Homoharringtonine (HHT), an alkaloid isolated from *C. harringtonia*, is the most widely studied anti-cancer compound and has been approved by the US FDA for treating patients with chronic myeloid leukemia (CML) [15]. *Catharanthus roseus* has been extensively investigated, leading to the isolation of two active alkaloids: the vinca alkaloids vinblastine and vincristine. These two compounds were the first plant-derived anticancer agents approved by the US FDA and commonly used to treat various types of cancer, including leukemia, breast cancer, lymphomas and lung cancer [16].

Knowledge of the anticancer effect of Phit-Sanat, particularly against leukemia, remains limited. This study presents a new report focusing on the activity of fractional extracts and purified compounds derived from active fractional extracts on leukemic cell lines.

Leukemia, a hematopoietic stem cell disorder, is characterized by the accumulation of abnormal blast cells and leukocytes in the peripheral blood and bone marrow. According to the GLOBOCAN 2022 database, published by the International Agency for Research on Cancer (IARC) and disseminated as Cancer Today on the Global Cancer Observatory, leukemia ranked 13th among the most frequently diagnosed cancers and 10th among the leading cause of cancer-related death worldwide. In 2022, more than 480,000 new cases and 305,000 deaths were estimated. The incidence of leukemia continues to rise each year [17, 18]. Leukemia is classified based on cell maturity (acute and chronic) and leukocyte lineages (myelocyte and lymphocyte). Wilms’ tumor 1 (WT1) protein, encoded by the *WT1* gene, plays a crucial role in leukemogenesis. Although the *WT1* gene was initially identified as a tumor suppressor, it can also function as an oncogene in leukemia and various solid tumors. Overexpression of the WT1 protein has been observed in leukemia, particularly in acute myeloblastic leukemia (AML) and acute lymphoblastic leukemia (ALL). Furthermore, WT1 mRNA levels have been found to increase significantly with the progression of chronic myelocytic leukemia (CML) and myelodysplastic syndrome [19–21]. Previous studies have also shown that the high WT1 protein expression is associated with poor prognosis and a high risk of AML [22–25].

The antiproliferative effects of Phit-Sanat root extract in leukemia have not been elucidated. This study aimed to investigate and compare the cytotoxicity of Phit-Sanat from 12 different sources in Thailand. Active compounds from the most effective crude fractional extract (*n*-hexane, ethyl acetate, or ethanol) were purified, identified, and analyzed for their anti-leukemic activity.

## Materials and methods

### Chemical materials

In this study, 3-(4,5-Dimethylthiazol-2-yl)−2,5-diphenyltetrazolium bromide (MTT) was purchased from Sigma-Aldrich (St. Louis, MO, USA). RPMI-1640 medium, IMDM medium, and penicillin–streptomycin were purchased from GIBCO Invitrogen™ (Grand Island, NY, USA). Fetal bovine serum (FBS) was obtained from Biochrom AG (Berlin, Germany). Protein markers, 30% acrylamide/bis solution, 1 M Tris-HCl, pH 8 solution, and 0.5 M Tris-HCl, pH 6.8 solution were purchased from Bio-Rad Laboratories (Richmond, CA, USA). Ethanol (EtOH), ethyl acetate (EtOAc), *n*-hexane (Hex), and dimethyl sulfoxide (DMSO) were purchased from Labscan (Dublin, Ireland). Silica gel 60 was purchased from Merck (Darmstadt, Germany).

### Plant materials

*S. exigua* were collected from 12 locations in Thailand, including Bangkok, Nonthaburi, Lopburi, Phitsanulok, Phuket, Chiang Mai, Chaiyaphum, and Nong Bua Lumphu provinces during April 2020. All plant materials were identified and authenticated by Angkhana Inta (Chiang Mai University), Sukanda Chaiyong, Trinnakorn Katekulaphan, and Methee Rungrojsakul (Chandrakasem Rajabhat University, Bangkok, Thailand). Voucher specimens (WP6610, WP6611, and WP6612) have been deposited at the Herbarium, Queen Sirikit Botanical Garden. The dried roots of *S. exigua* from all 12 sources were ground into a fine powder using a Moulinex mixer blender and subsequently extracted with Hex (500 mL × 3 times), EtOAc (500 mL × 3 times), and EtOH (500 mL × 3 times), respectively. The resulting Hex, EtOAC, and EtOH extracts were filtered and concentrated under reduced pressure, yielding a total of 36 extracts. These crude fractional extracts were then evaluated for their anti-leukemic activity.

### Column chromatography

The effective crude fractional extract was purified with column chromatography. Silica gel grade 60 was used as the stationary phase and packed in a column. The crude fractional extract (5.0 g) was added to the top of the silica gel. Different ratios of Hex and EtOAc were used as the mobile phase to separate the compounds by increasing the polarity of solvents with the proportion of Hex : EtOAc = 100 : 0 to 0 : 100. Fractions were collected in test tubes (8 mL/tubes) and we determined compounds in each fraction with thin layer chromatography (TLC). Fractions with similar TLC patterns were pooled together, observed using TLC, and characterized in terms of their chemical structures using 500 MHz nuclear magnetic resonance (NMR) spectroscopy (Bruker, Fällanden, Switzerland) at the Faculty of Science, Chiang Mai University, or with a JEOL (Tokyo, Japan) JNM-ECA 500 MHz spectrometer at the Department of Materials and Life Sciences, Sophia University, Tokyo, Japan. Using electrospray ionization–mass spectrometry (ESI-MS) spectra with time-of-flight (TOF) detection for high-resolution measurements, spectra were recorded on a JEOL JMS-T100LC instrument at Sophia University. *S. exigua* crude fractional extracts and purified compounds were stored at − 20 °C. The crude fractional extracts and compounds were dissolved in DMSO to obtain the working concentration (25 mg/mL) and stored at − 20 °C for later use.

### Cells and cell culture conditions

Leukemic cell lines included KG-1a (leukemic stem cell-like cell line with stem cell population) and EoL-1 (human promyelocytic leukemia cell line). Both cell Lines used in this study had passage numbers ranging from 5 to 15. Peripheral blood mononuclear cells (PBMCs) were isolated from healthy donors using Ficoll-Hypaque density-gradient centrifugation with Lymphoprep™ solution (Axis-Shield, Oslo, Norway). KG-1a cells were cultured in IMDM medium supplemented with 20% FBS, 100 units/mL penicillin, and 100 µg/mL streptomycin. EoL-1 and PBMCs were cultured in RPMI-1640 medium supplemented with 10% FBS, 1mM L-glutamine, 100 units/mL penicillin, and 100 µg/mL streptomycin. All cell Lines were maintained at 37 °C in an atmosphere of 95% humidity and 5% CO_2_. The use of human PBMCs in this study was approved by the Human Research Ethics Unit of the Faculty of Associated Medical Sciences, Chiang Mai University, Chiang Mai, Thailand. The study code was No. 196/2022, and the date of approval was June 27, 2022.

### Cytotoxicity determination by MTT assay

1 $$\%\mathrm{Cell}\;\mathrm{viability}\;=\;\frac{\mathrm{Mean}\;\mathrm{absorbance}\;\mathrm{in}\;\mathrm{test}\;\mathrm{well}}{\mathrm{Mean}\;\mathrm{absorbance}\;\mathrm{in}\;\mathrm{vehicle}\;\mathrm{control}\;\mathrm{well}}\;\times\;100$$

The MTT (3-(4,5-dimethylthiazol-2-yl)−2,5-diphenyltetra-zolium bromide) assay was used to determine the cytotoxicity of *S. exigua* crude fractional extracts and active compounds on leukemic cells and PBMCs. KG-1a (2.0 × 10^5^ cells/mL), EoL-1 (3.0 × 10^5^ cells/mL), and PBMCs (1.0 × 10^6^ cells/mL) were treated with various concentrations of *S. exigua* extracts and active compounds for 48 h. Then, 100 µL of the medium was removed, and 15 µL of MTT dye solution (5 mg/mL) was added. The cells were further incubated for 4 h. Subsequently, 200 µL of DMSO was added to each well, then mixed gently to dissolve the purple formazan crystals. The optical density was measured using an ELISA plate reader at 578 nm, with a reference wavelength of 630 nm. The percentage of surviving cells was calculated using the following Eq. (1):

The average percentage of Living cells at each concentration, obtained from 3-independent experiments, was plotted as a dose-response curve. The IC_50_ (50% inhibitory concentration) was defined as the concentration required to inhibit 50% of cell growth compared to the untreated control.

### Trypan blue exclusion assay

Cell proliferation was measured using the trypan blue exclusion method. Cells were treated with crude fractional extracts and active compounds under different conditions as indicated and incubated at 37 °C. Then, the cells were mixed with 0.4% trypan blue dye and counted under a light microscope using an Invitrogen™ Countess™ 3 Automated Cell Counter (Thermo Fisher Scientific, Waltham, MA, USA). All experiments were performed in triplicate.

### Flow cytometry analysis of cell cycle distribution

KG-1a and EoL-1 cells were treated with various concentrations of *S. exigua* crude fractional extract and active compounds for different time points. After incubation, the cells were harvested and washed twice with cold PBS, pH 7.4. They were then fixed in 66% ice-cold ethanol, washed again with cold PBS, pH 7.4, and incubated with 1× propidium iodide and RNase staining solution (Abcam, Cambridge, UK) at room temperature for 20–30 min in the dark. Cell cycle distribution was assessed using an LSRII flow cytometer (BD biosciences, USA).

### Flow cytometry analysis of apoptosis

KG-1a and EoL-1 cells were treated with *S. exigua* extracts and active compounds under various treatment conditions. Apoptosis was analyzed using the FITC Annexin V apoptosis detection kit with PI (BioLegend, San Diego, CA, USA) according to the manufacturer’s protocols. Briefly, the treated cells were harvested, washed with cold PBS, pH 7.4 and resuspended in Annexin V binding buffer. The resuspended cells were then incubated with 5 µL of FITC Annexin V and 10 µL of PI solution for 15 min at room temperature in the dark, followed by the addition of 400 µL of Annexin V binding buffer. Apoptosis was assessed using an LSRII flow cytometer.

### Western blot analysis

KG-1a and EoL-1 cells were cultured with the crude fractional extract or active compounds under varying time and dose conditions as indicated and incubated at 37 °C in a humidified incubator. After incubation, the cells were harvested, and total protein was extracted using RIPA buffer (50 mM Tris-HCl, 150 mM NaCl, 1% Triton X-100, 0.5 mM EDTA, 0.1% SDS, and a protease inhibitor cocktail). Protein concentration was measured using Pierce™ BCA Protein Assay Kit (Thermo Fisher Scientific, Waltham, MA, USA). Then, 30–50 µg of each sample was loaded, and protein was separated using 10−15% SDS-PAGE, followed by transferred to a PVDF membrane. The membranes were first blocked with 5% skim milk in Tris-buffered saline with 0.1%Tween-20 (TBST) and then incubated with primary antibodies while gently shaking. The primary antibodies used were as follows: rabbit monoclonal anti-CDK4 (D9G3E) IgG, rabbit monoclonal anti-cleaved caspase-3 (Asp175)(5A1E) IgG, and rabbit monoclonal anti-WT1 IgG (1:1,000, 4 °C, overnight; Cell Signaling Technology, Danvers, MA, USA). Additionally, rabbit polyclonal anti-human GAPDH IgG (1:16,000, RT, 1 h; Santa Cruz Biotechnology, CA, USA) was used as a loading control. Proteins detection was performed using TBST containing a 1:10,000 dilution of goat anti-rabbit IgG secondary antibody (Bio-Rad, USA) and an anti-biotin, HRP-linked antibody (Cell Signaling Technology, Danvers, MA, USA). Proteins were then visualized using the chemiluminescence technique with Supersignal™ West Femto Maximum Sensitivity Substrate (Thermo Fisher Scientific, Waltham, MA, USA) and photographed with a myECL™ Imager (Thermo Fisher Scientific, Waltham, MA, USA). Protein band densitometric analysis was performed using Quantity One 1-D Analysis software (Bio-Rad, USA). The band density of each targeted protein was normalized to that of GAPDH. All experiments were performed in triplicate.

### Proteomics analysis

Leukemic cells were treated with the active compound and DMSO (as a vehicle control) for 24 h. After treatment, the cells were harvested, followed by extraction and centrifugation. Cell pellet was resuspended in 0.1 mL of RIPA buffer containing a protease inhibitor and PhosSTOP™ phosphatase inhibitor cocktail (Sigma-Aldrich, St. Louis, MO, USA). The protein concentration was then measured using the BCA method. Based on the protein concentration, 100 µg of total protein was used for proteomic analysis at the UC Davis Proteomic Core Facility, UC Davis, Sacramento, CA, USA.

The acquired raw data were then analyzed for protein quantification. Gene ontology (GO) and Kyoto Encyclopedia of Genes and Genomes (KEGG) pathway enrichment analysis of differential proteins were performed using the online software DAVID (https://david.ncifcrf.gov/). The network map of differential proteins was created using the SRplot online tool (https://www.bioinformatics.com.cn/en). Proteins were considered significantly differentially expressed when *p*-value for differential expression was less than 0.05 (*p* < 0.05).

### Statistical analysis

Results are presented as the mean ± SD (standard deviation) from three independent experiments. Statistical analysis was performed using a one-way ANOVA followed by the LSD post-hoc test. The level of statistical significance (*p* value) was less than 0.05 (*p < 0.05), 0.01 (**p < 0.01), and 0.001 (***p < 0.001).

## Results

### Yield of extracts

Phit-Sanat from 12 locations extracted from Hex, EtOAc, and EtOH, provided 36 fractional extract samples with varying %yields. The EtOAc fractional extracts exhibited the highest %yields, ranging from 8.13 ± 0.06 to 18.80 ± 0.07%, followed by the EtOH fractional extracts (2.40 ± 0.05 to 5.80 ± 0.10%) and Hex fractional extracts (1.64 ± 0.06 to 4.42 ± 0.03%), as shown in Table 1.

**Table 1:** % Yields of crude fractional extracts from 12 locations of *S. exigua*

Crude fractional extract (Province)	Yield (%w/w)		
	Hex	EtOAc	EtOH
001 (Bangkok 1)	2.79 ± 0.09	12.89 ± 0.09	2.40 ± 0.05
002 (Bangkok 2)	2.71 ± 0.07	13.34 ± 0.07	3.74 ± 0.07
003 (Bangkok 3)	2.30 ± 0.06	12.86 ± 0.09	4.19 ± 0.03
004 (Bangkok 4)	3.05 ± 0.06	13.88 ± 0.10	3.15 ± 0.07
005 (Nonthaburi)	2.92 ± 0.06	12.71 ± 0.06	3.33 ± 0.07
006 (Phitsanulok)	2.41 ± 0.09	10.92 ± 0.05	2.92 ± 0.04
007 (Lopburi)	3.47 ± 0.05	8.13 ± 0.06	3.29 ± 0.03
008 (Phuket)	4.42 ± 0.03	9.43 ± 0.06	4.22 ± 0.08
009 (Chiang Mai)	3.16 ± 0.08	9.54 ± 0.09	4.16 ± 0.06
010 (Chaiyaphum)	3.88 ± 0.09	15.85 ± 0.05	4.53 ± 0.06
011 (Nong Bua Lamphu 1)	1.64 ± 0.06	17.29 ± 0.08	4.27 ± 0.04
012 (Nong Bua Lamphu 2)	2.87 ± 0.08	18.80 ± 0.07	5.80 ± 0.10

Results expressed as mean ± SD

### Cytotoxicity screening of crude fractional extracts from *S. exigua* in KG-1a and EoL-1 leukemic cells, and PBMCs

To determine the cytotoxicity of crude fractional extracts from *S. exigua* in the KG-1a and EoL-1 leukemic cell Lines, cells were treated with various concentrations of these extracts and assessed using the MTT assay. EtOAc No. 005 (from Nonthaburi), No. 006 (from Phitsanulok), No. 010 (from Chaiyaphum), and No. 011 (from Nong Bua Lamphu 1) exhibited significant cytotoxic effects in all leukemic cell lines, as shown in Table 2.

**Table 2:** IC_50_ values (µg/mL) of crude fractional extracts from 12 sources of *S. exigua* in KG-1a and EoL-1 leukemic cells, and PBMCs

Crude fractional extract (Province)	IC_50_ value (mean ± SD, µg/mL)		
	KG-1a	EoL-1	PBMCs
001 (Bangkok 1)			
* n*-Hexane	> 100	63.40 ± 1.44	ND
EtOAc	73.39 ± 5.94	34.80 ± 1.14	ND
EtOH	> 100	> 100	ND
002 (Bangkok 2)			
* n*-Hexane	> 100	67.30 ± 4.02	ND
EtOAc	62.74 ± 10.54	28.85 ± 1.21	ND
EtOH	> 100	> 100	ND
003 (Bangkok 3)			
* n*-Hexane	> 100	40.35 ± 3.38	ND
EtOAc	59.86 ± 7.33	23.25 ± 1.82	ND
EtOH	> 100	> 100	ND
004 (Bangkok 4)			
* n*-Hexane	> 100	67.71 ± 3.80	ND
EtOAc	56.30 ± 9.11	25.23 ± 0.77	ND
EtOH	> 100	> 100	ND
005 (Nonthaburi)			
* n*-Hexane	> 100	71.10 ± 0.99	ND
EtOAc	61.01 ± 4.07	22.32 ± 1.43	69.61 ± 6.22
EtOH	> 100	> 100	ND
006 (Phitsanulok)			
* n*-Hexane	> 100	39.95 ± 0.62	ND
EtOAc	64.75 ± 4.19	24.80 ± 2.55	74.73 ± 9.53
EtOH	> 100	> 100	ND
007 (Lopburi)			
* n*-Hexane	> 100	72.62 ± 1.80	ND
EtOAc	72.32 ± 2.03	35.15 ± 1.18	ND
EtOH	> 100	> 100	ND
008 (Phuket)			
* n*-Hexane	> 100	> 100	ND
EtOAc	91.06 ± 5.61	42.38 ± 2.24	ND
EtOH	> 100	> 100	ND
009 (Chiang Mai)			
* n*-Hexane	> 100	73.34 ± 3.78	ND
EtOAc	91.90 ± 10.44	44.06 ± 3.47	ND
EtOH	> 100	> 100	ND
010 (Chaiyaphum)			
* n*-Hexane	> 100	62.74 ± 3.68	ND
EtOAc	29.52 ± 3.36*	13.19 ± 1.10*	47.77 ± 1.10
EtOH	> 100	> 100	ND
011 (Nong Bua Lamphu 1)			
* n*-Hexane	> 100	60.20 ± 2.82	ND
EtOAc	57.04 ± 5.50	23.77 ± 0.52	83.34 ± 8.58
EtOH	> 100	> 100	ND
012 (Nong Bua Lamphu 2)			
* n*-Hexane	> 100	37.57 ± 1.95	ND
EtOAc	52.95 ± 7.38	25.35 ± 1.55	ND
EtOH	> 100	> 100	ND

Results expressed as mean ± SD of three independent experiments

*ND* Not determined

*Good cytotoxicity in each cell line

Among the 36 crude fractional extracts, EtOAc No. 010 exhibited strong cytotoxicity in vitro in KG-1a and EoL-1 cells, with IC_50_ values of 29.52 ± 3.36 and 13.19 ± 1.10 µg/mL, respectively. Moreover, EtOAc No. 010 showed low toxicity in normal PBMCs at 3.125–25 µg/mL, while high concentration exhibited cytotoxicity, with an IC_50_ value of 47.77 ± 1.10 µg/mL. Therefore, EtOAc No. 010 and its concentration at the IC_20_ values for both cell lines were selected for further investigations in KG-1a and EoL-1 leukemic cells.

### Purification and identification of active compounds from *S. exigua*

**Fig. 1:**
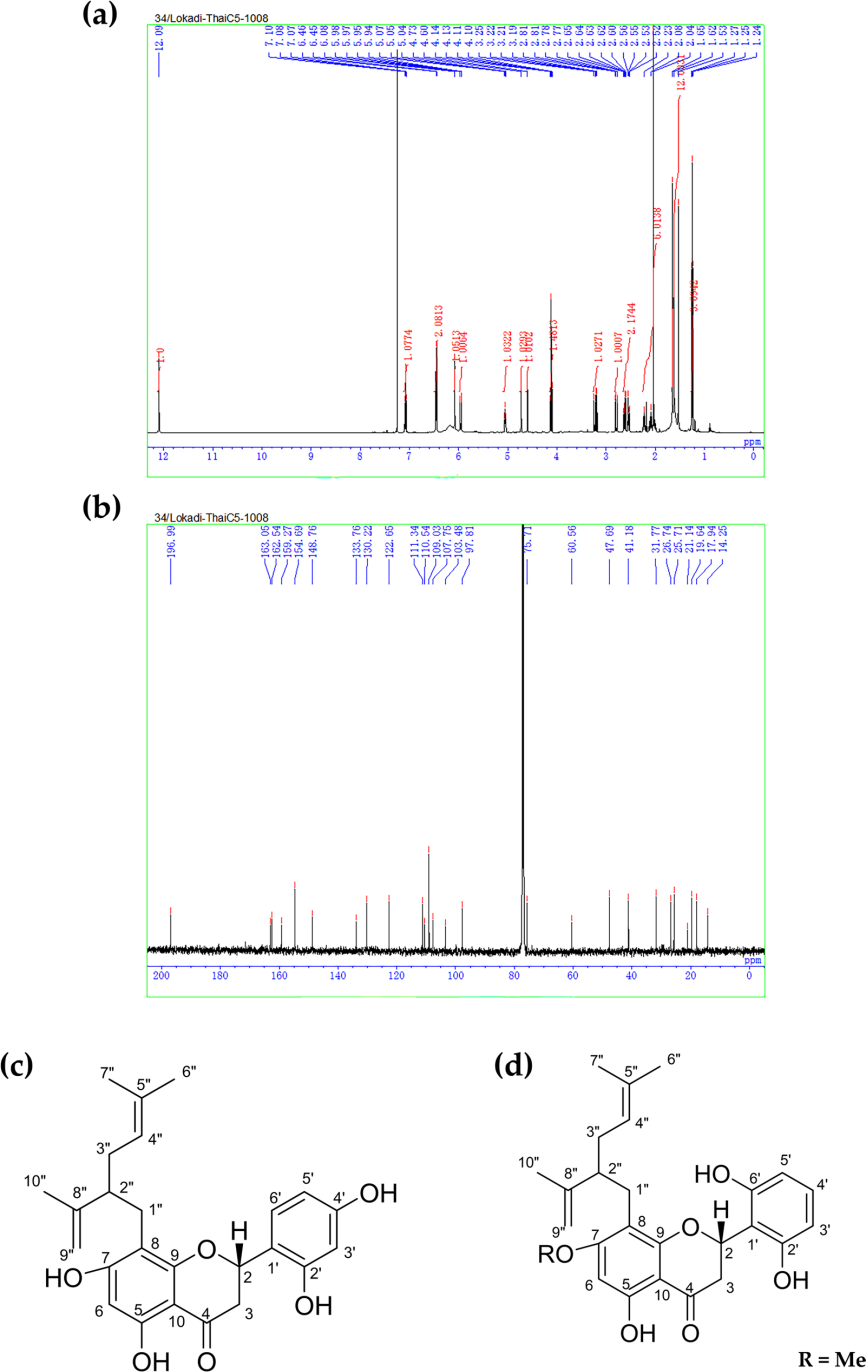
Active compounds of *S. exigua*. Identification of sophoraflavanone G (SG) includes **a**^1^H- and **b**^13^C-NMR spectra of SG in CDCl_3_, and **c** chemical structure of SG. **d** The chemical structure of exiguaflavanone B (EGF-B)

EtOAc No. 010 (5 g), an active crude fractional extract, was subjected to silica gel column chromatography to separate its purified compounds. Among 18 obtained compounds, one purified compound, a yellowish-orange powder-like substance, was isolated with the highest yield (10.42%). The ^1^H- (Fig. 1a), ^13^C- (Fig. 1b), HMBC (Supplementary Fig. 1a), HMQC (Supplementary Fig. 1b), and HSQC (Supplementary Fig. 1c) NMR spectra were obtained for this purified compound, which was identified as sophoraflavanone G (SG) based on comparison with published data [26, 27]. The characterizations of the active compound are as follows.

Sophoraflavanone G: yellowish orange powder-like substance; ^1^H NMR (CDCl_3_, 500 MHz): δ 12.09 (1H, s), 7.08 (1H, t, *J* = 10.0 Hz), 6.40 (2 H, d, *J =* 5.0 Hz), 6.08 (1H, s), 5.96 (2 H, dd, *J* = 15.0, 5.0 Hz), 5.05 (1H, m), 4.73 (1H, 1H, s), 4.60 (1H, s), 4.12 (1H, q, *J* = 5.0 Hz), 3.22 (1H, dd, *J* = 15.0, 10.0 Hz), 2.80 (1H, dd, *J* = 15.0, 5.0 Hz), 2.65–2.52 (1H, m), 2.25-2.00 (6 H, m), 1.65 (3 H, s), 1.62 (3 H, s), 1.53 (3 H, s), 1.25 (3 H, t, *J* = 5.0 Hz); ^13^C NMR (CDCl_3_, 125 MHz) δ 197.0, 163.1, 162.5, 159.3, 154.7, 148.8, 133.8, 130.2, 122.7, 111.3, 110.5, 109.0, 107.8, 103.5, 97.8, 75.7, 60.6, 47.7, 41.2, 31.8, 26.7, 25.7, 21.1, 19.6, 17.9, 14.3; ESI-HRMS (*m*/*z*) calcd for C_25_H_27_O_6_ [M−H]^−^ 423.1808, found 423.1808.

SG exerts antibacterial activity by reducing the fluidity of the bacterial membrane [10]. It also has anti-malarial, anti-allergic and anti-cancer properties [28]. Furthermore, exiguaflavanone B (EGF-B) (Fig. 1 d), another important active compound, was isolated from *S. exigua* and provided from the Department of Biochemistry, Faculty of Medicine, Chiang Mai University, Chiang Mai, Thailand. According to previous studies, EGF-B possesses potent antibacterial activity [29] and has a similar carbon skeleton to a frullanolide compound, which exhibited inhibitory effects against MDA-MB-231, MDA-MB-468, and MCF-7 breast cancer cells [30]. In 2023, Arjsri et al. found that EGF-B could inhibit the proliferation and metastasis of NSCLC cells [31]. Therefore, EGF-B and SG were further investigated for their anti-leukemic activity.

### Cytotoxicity of active compounds from *S. exigua* in KG-1a and EoL-1 leukemic cells and PBMCs

**Fig. 2:**
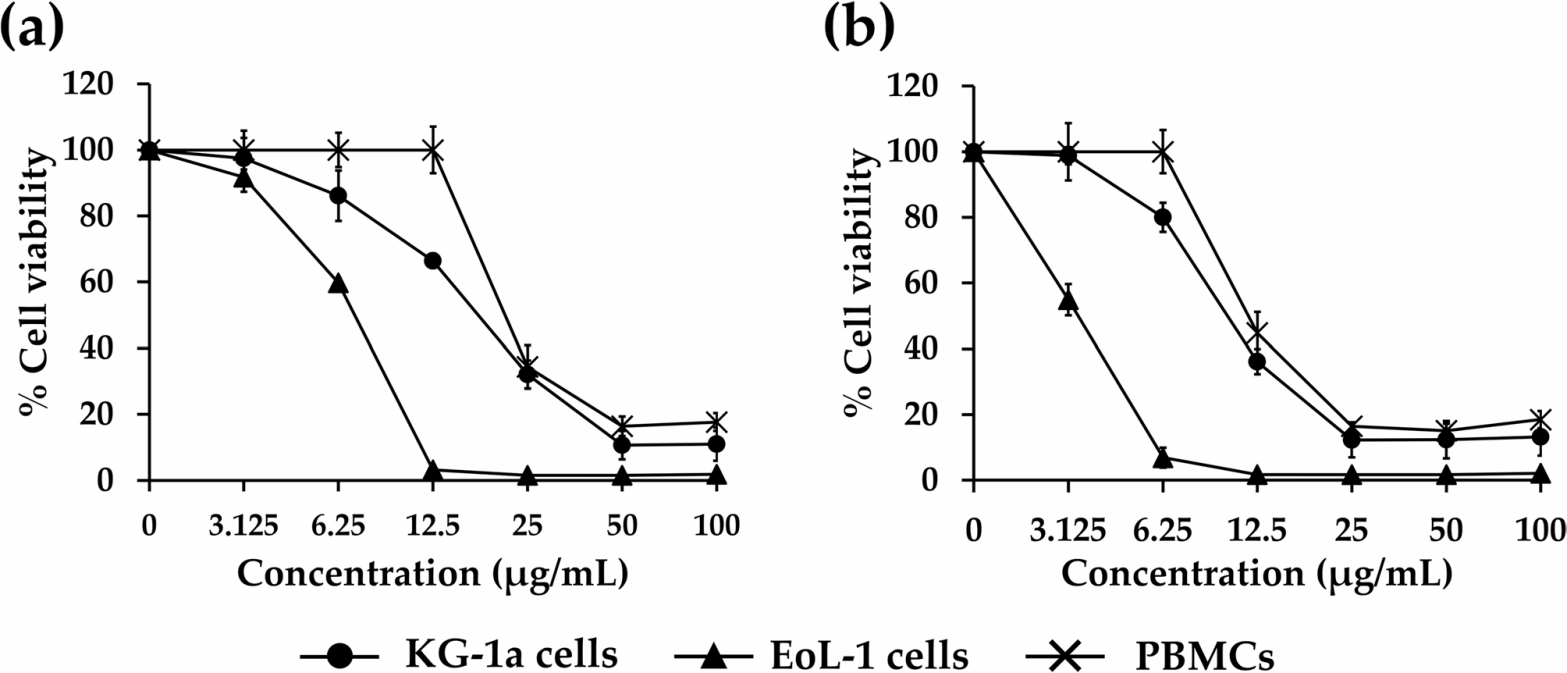
Cytotoxicity of active compounds from *S. exigua* in leukemic cell lines (KG-1a and EoL-1 cells) and PBMCs. KG-1a, EoL-1, and PBMCs were treated with **a** EGF-B and **b** SG for 48 h. Cell viability was assessed by the MTT assay. Results are expressed as mean± SD of three independent experiments.

EGF-B and SG were then analyzed for their cytotoxicity in leukemic cell lines (KG-1a and EoL-1) and PBMCs using the MTT assay. These compounds inhibited the proliferation of both leukemic cell lines. EGF-B exhibited cytotoxicity in the KG-1a and EoL-1 leukemic cells, with IC_50_ values of 18.52 ± 0.71 and 7.32 ± 0.13 µg/mL, respectively. SG also demonstrated cytotoxic effects in these leukemic cells with IC_50_ values of 10.54 ± 0.28 and 3.40 ± 0.35 µg/mL, respectively (Fig. 2). Additionally, EGF-B and SG showed cytotoxic effects against PBMCs, with IC_50_ values of 22.53 ± 1.47 and 12.07 ± 1.06 µg/mL, respectively. These compounds exhibited significantly higher cytotoxicity in KG-1a and EoL-1 leukemic cell lines than in PBMCs. The concentrations at the IC_20_ (20% inhibitory concentration or 80% viable cells) of both compounds were used to investigate changes in protein expression in both leukemic cell lines after treatment.

### KG-1a and EoL-1 leukemic cell viability after treatment with crude fractional extract and active compounds of *S. exigua*

**Fig. 3:**
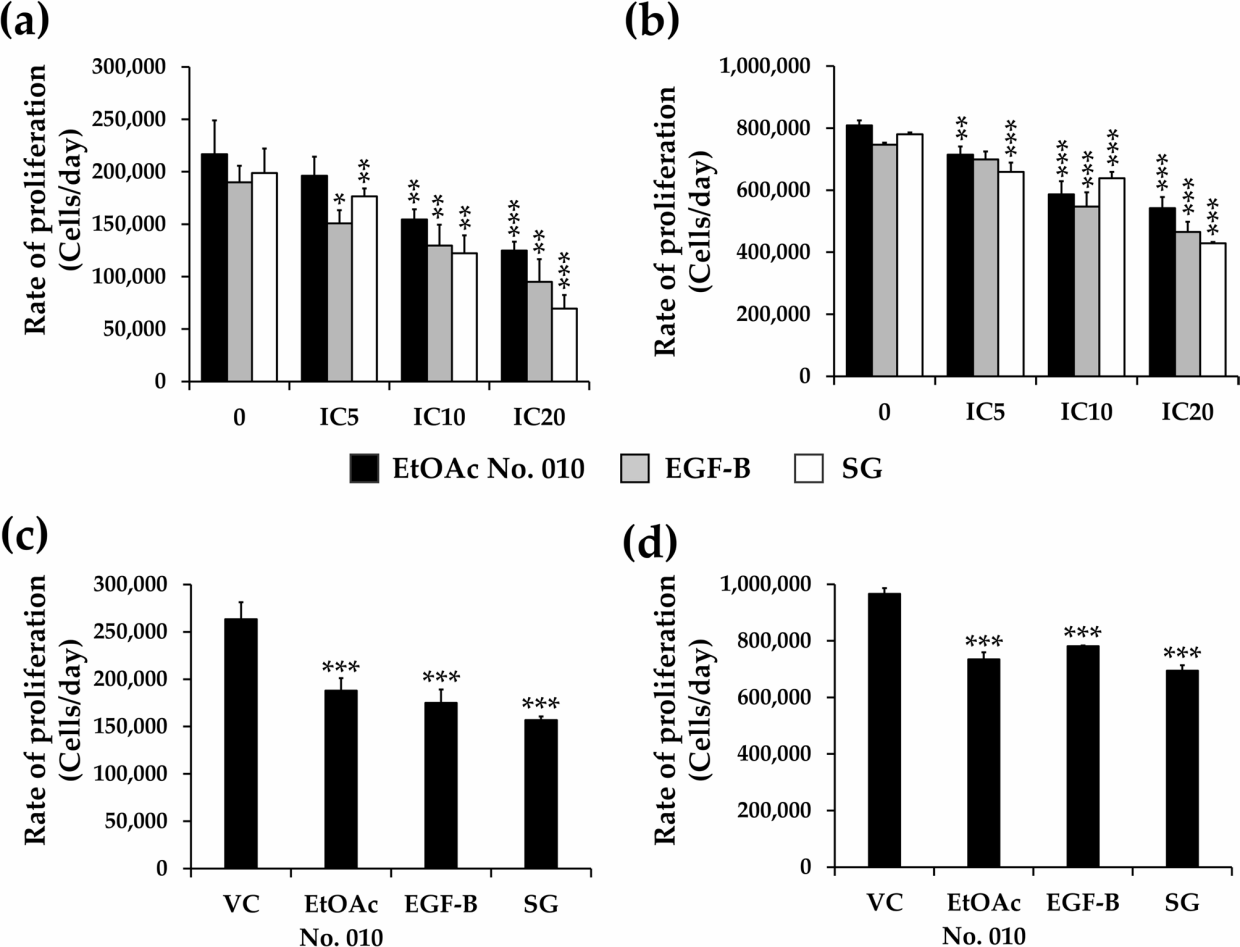
Quantification of cell viability. **a** KG-1a and **b** EoL-1 cells were treated with various doses (IC_5_, IC_10_, and IC_20_) of EtOAc No. 010, EGF-B, and SG for 48 h. Furthermore, **c** KG-1a and **d** EoL-1 were treated with EtOAc No. 010, EGF-B, and SG at IC_20_ concentrations for 24, 48, and 72 h. The number of cells was counted using trypan blue exclusion assay. Results expressed as mean± SD of three independent experiments. Asterisks (*) denote significant differences from the vehicle control (* *p*< 0.05, ** *p*< 0.01, and *** *p*< 0.001)

To determine the inhibitory effect of the crude fractional extract and active compounds of *S. exigua* on leukemic cell viability, KG-1a and EoL1 cells were treated with EtOAc crude fractional extract No. 010 and its compounds at various concentrations for 48 h, or at the IC_20_ concentration for 24, 48, and 72 h. The viable cell number was quantified using the trypan blue assay. As shown in Fig. 3, the EtOAc No. 010 extract and both active compounds inhibited the viability of leukemic cells in a dose- and time-dependent manner.

### Effects of crude fractional extract and active compounds of *S. exigua* on cell cycle distribution

**Fig. 4:**
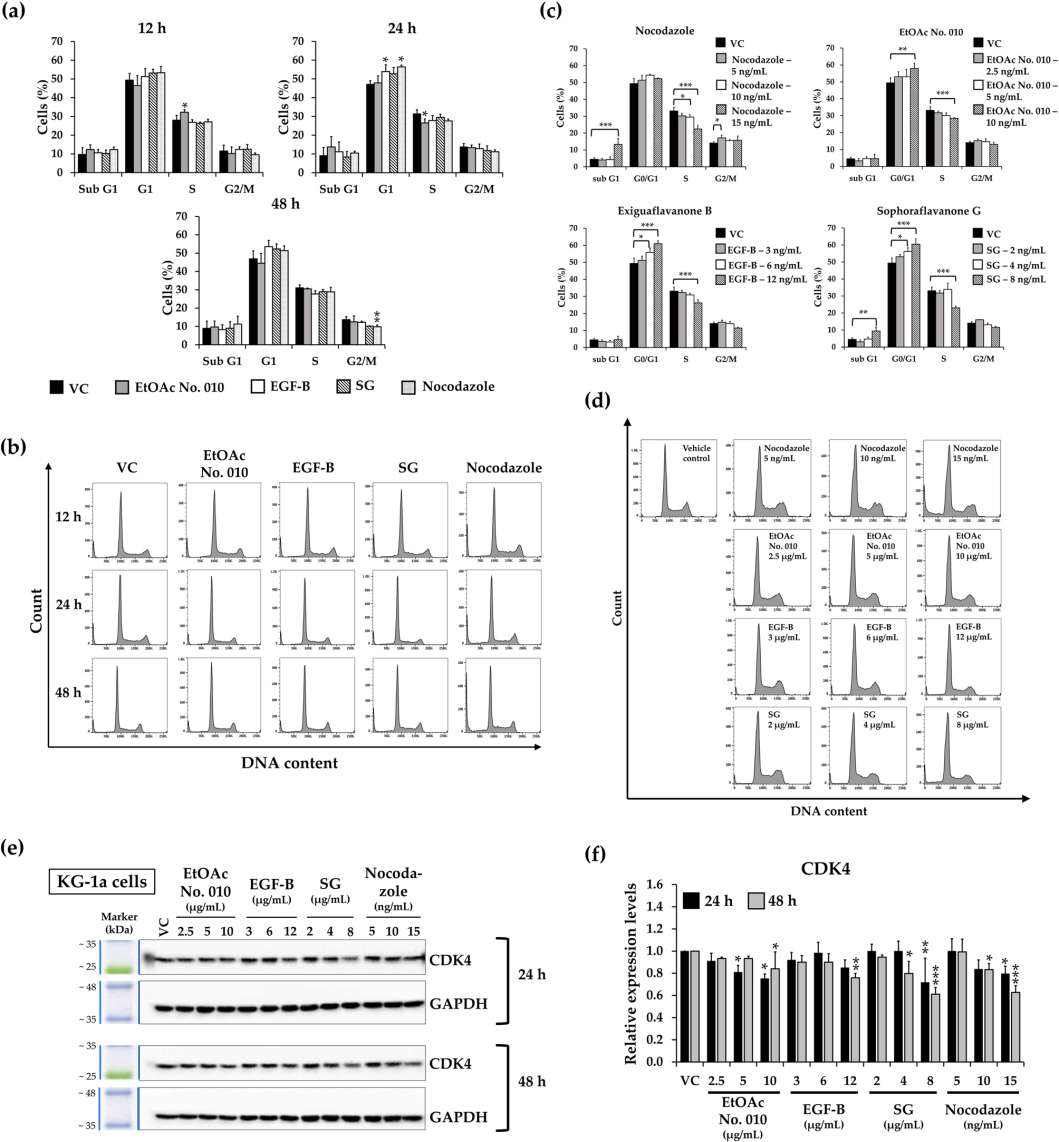
Effect of the crude fractional extract of *S. exigua* (EtOAc No. 010) and the active compounds (EGF-B and SG) on the cell cycle distribution of KG-1a cells. **a**,**b** KG-1a cells were treated with EtOAc No. 010, EGF-B, SG, and nocodazole (as a positive control) at IC_20_ concentrations for 12, 24, and 48 h. **c**,**d** Additionally, KG-1a cells were treated with various concentrations of EtOAc No. 010, EGF-B, SG, and nocodazole for 24 h. **e**,**f** Western blot analysis of CDK4 expression in KG-1a cells after treatment with the crude fractional extract of *S. exigua* (EtOAc No. 010) and the active compounds (EGF-B and SG) for 24 and 48 h. Band intensity was quantified using a scanning densitometer, with GAPDH as the internal control. **a**,**c**,**f** Quantification of three independent replicates (n = 3), and **b**,**d**,**e** representative images are shown. Data are expressed as the mean ± SD from three independent experiments. *p< 0.05, **p< 0.01, and ***p< 0.001 vs. control group

**Fig. 5:**
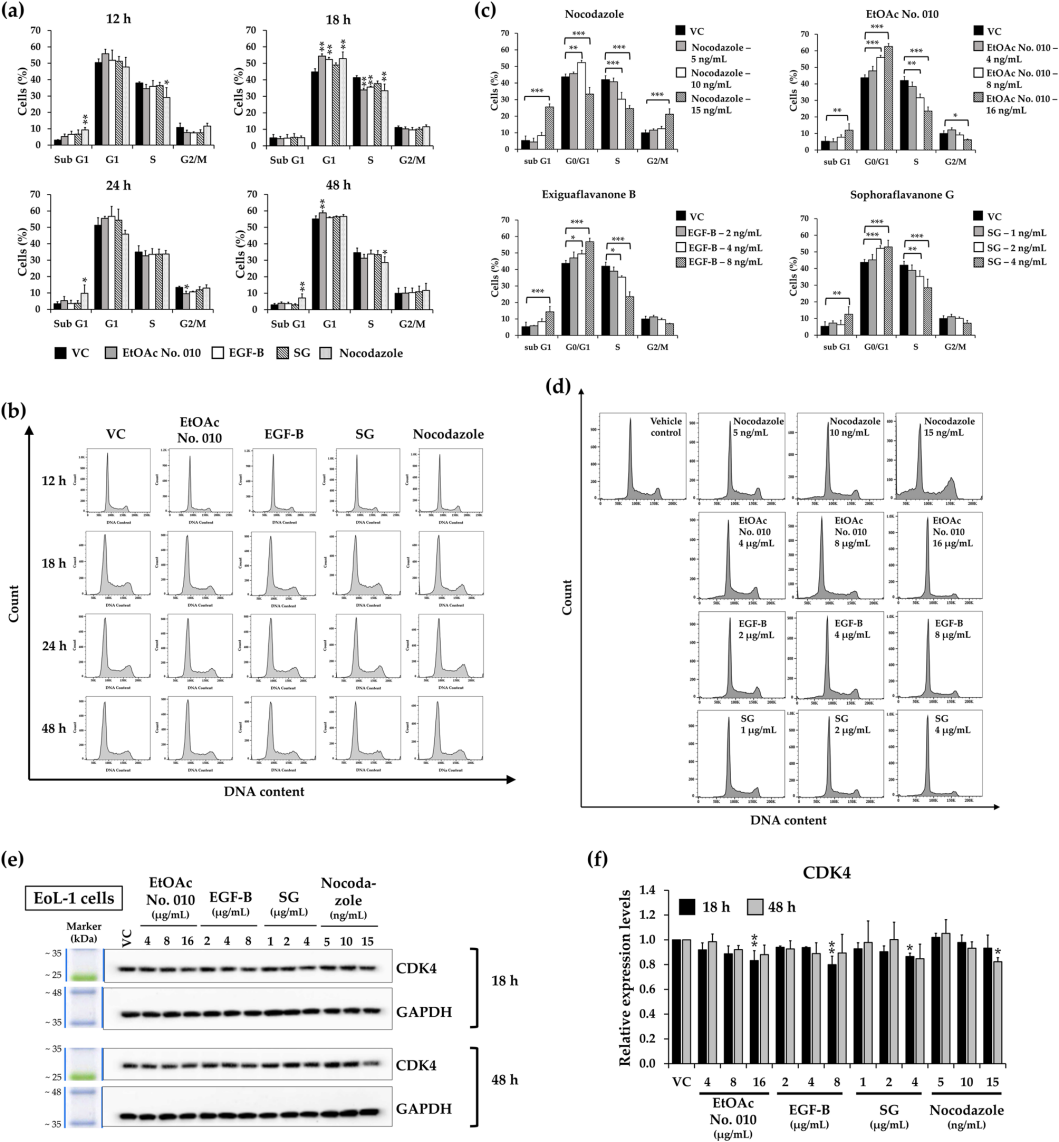
Effect of the crude fractional extract of *S. exigua* (EtOAc No. 010) and the active compounds (EGF-B and SG) on the cell cycle distribution of EoL-1 cells.** a**,** b **EoL-1 cells were treated with EtOAc No. 010, EGF-B, SG, and nocodazole (as positive control) at IC_20_ concentrations for 12, 18, 24, and 48 h.** c**,** d **Additionally, EoL-1 cells were treated with various concentrations of EtOAc No. 010, EGF-B, SG, and nocodazole for 18 h.** e**,** f **Western blot analysis of CDK4 expression in EoL-1 cells after treatment with the crude fractional extract of *S. exigua* (EtOAc No. 010) and the active compounds (EGF-B and SG) for 18 and 48 h. Band intensity was quantified using a scanning densitometer, with GAPDH as the internal control.** a**,** c**,** f **Quantification of independent replicates (n = 3), and** b**,** d**,** e **representative images are shown. Data are expressed as the mean ± SD from three independent experiments. *p< 0.05, **p< 0.01, and ***p< 0.001 vs. control group

To evaluate the inhibitory effect of crude fractional extract and active compounds of *S. exigua* on leukemic cell proliferation, cell cycle distribution in KG-1a and EoL-1 leukemic cells was assessed using flow cytometry after treatment. Both cell lines were treated with IC_20_ values of EtOAc No. 010, EGF-B, SG, and nocodazole (as a positive control) for various time points. As shown in Figs. 4 and 5, at 24 h (KG-1a) and 18 h (EoL-1) of treatment, there was a statistically significant accumulation of cells in the G1 phase of the cell cycle. Furthermore, EtOAc No. 010, EGF-B, and SG significantly increased the accumulation of both KG-1a and EoL-1 cells at G1 phase in a dose-dependent manner, while decreasing S phase cell population. Interestingly, the highest concentration of SG and nocodazole induced cell accumulation in sub-G1 phase (less than 20% of cells) in both KG-1a and EoL-1 cells. However, at the highest concentrations, EtOAc No. 010 and EGF-B also increased the sub-G1 population in EoL-1 cell. Besides inducing cell cycle arrest, the crude fractional extract and active compounds also promoted leukemic cell death at higher concentrations. Nocodazole is an anti-mitotic agent that disrupts the microtubule assembly, imparing the formation of the metaphase spindle during cell division. This disruption induces G2/M phase arrest and apoptosis in tumor cells [32, 33]. According to the results, lower concentrations of nocodazole caused minor accumulation of KG-1a cells in the G1 phase, while significantly enhanced G1-phase cell accumulation in EoL-1 cells. When KG-1a and EoL-1 cell were treated with the highest concentration of nocodazole (approximately at IC_50_ values), an increase in the sub-G1 population and induction of G2/M arrest were observed in both cell lines.

The expression of CDK4, a protein involved in the transition from the G1 into the S phase, was analyzed by Western blotting. The result revealed that CDK4 was downregulated in a dose-dependent manner in KG-1a (Fig. 4e and f) and EoL-1 cells (Fig. 5e and f). These findings suggest that EtOAc No. 010 and the active compounds suppressed KG-1a and EoL-1 cell proliferation by inducing G1 phase arrest.

### Effects of crude fractional extract and active compounds of *S. exigua* on cell apoptosis

**Fig. 6:**
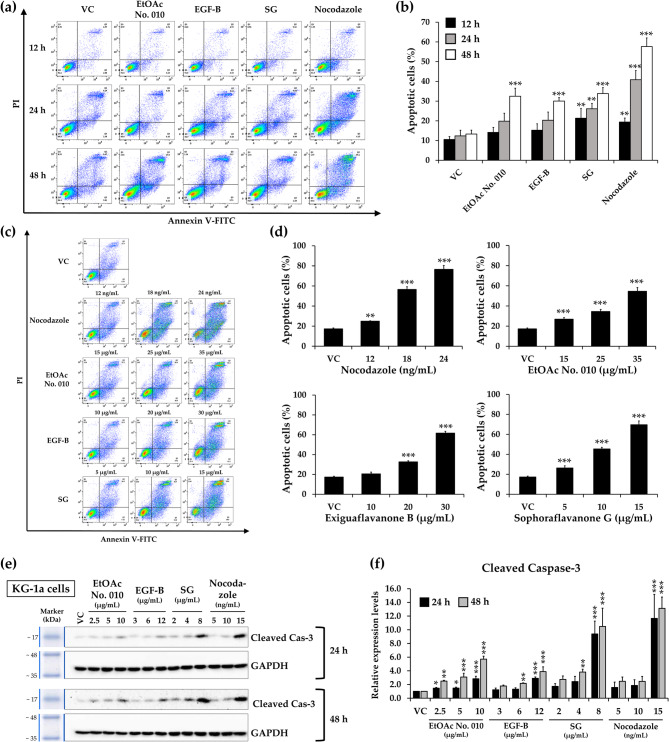
Effect of the crude fractional extract of *S. exigua* (EtOAc No. 010) and the active compounds (EGF-B and SG) on the induction of apoptosis in KG-1a cells.** a, b **KG-1a cells were treated with EtOAc No. 010, EGF-B, SG, and nocodazole (as positive control) at their IC_50_ concentrations for 12, 24, and 48 h.** c, d **Additionally, KG-1a cells were treated with various concentrations of EtOAc No. 010, EGF-B, SG, and nocodazole for 48 h.** e, f **Western blot analysis of cleaved caspase-3 in KG-1a cells following treatment with the crude fractional extract of *S. exigua*, EtOAc No. 010, and the active compounds (EGF-B and SG) for 24 and 48 h. Band intensity was determined using a scanning densitometer, with GAPDH serving as the internal control.** a, c, e **Representative images, and** b, d, f **quantification of three independent replicates (n = 3) are shown. Data are expressed as the mean ± SD from three independent experiments. *p< 0.05, **p< 0.01, and ***p< 0.001 vs. control group

**Fig. 7:**
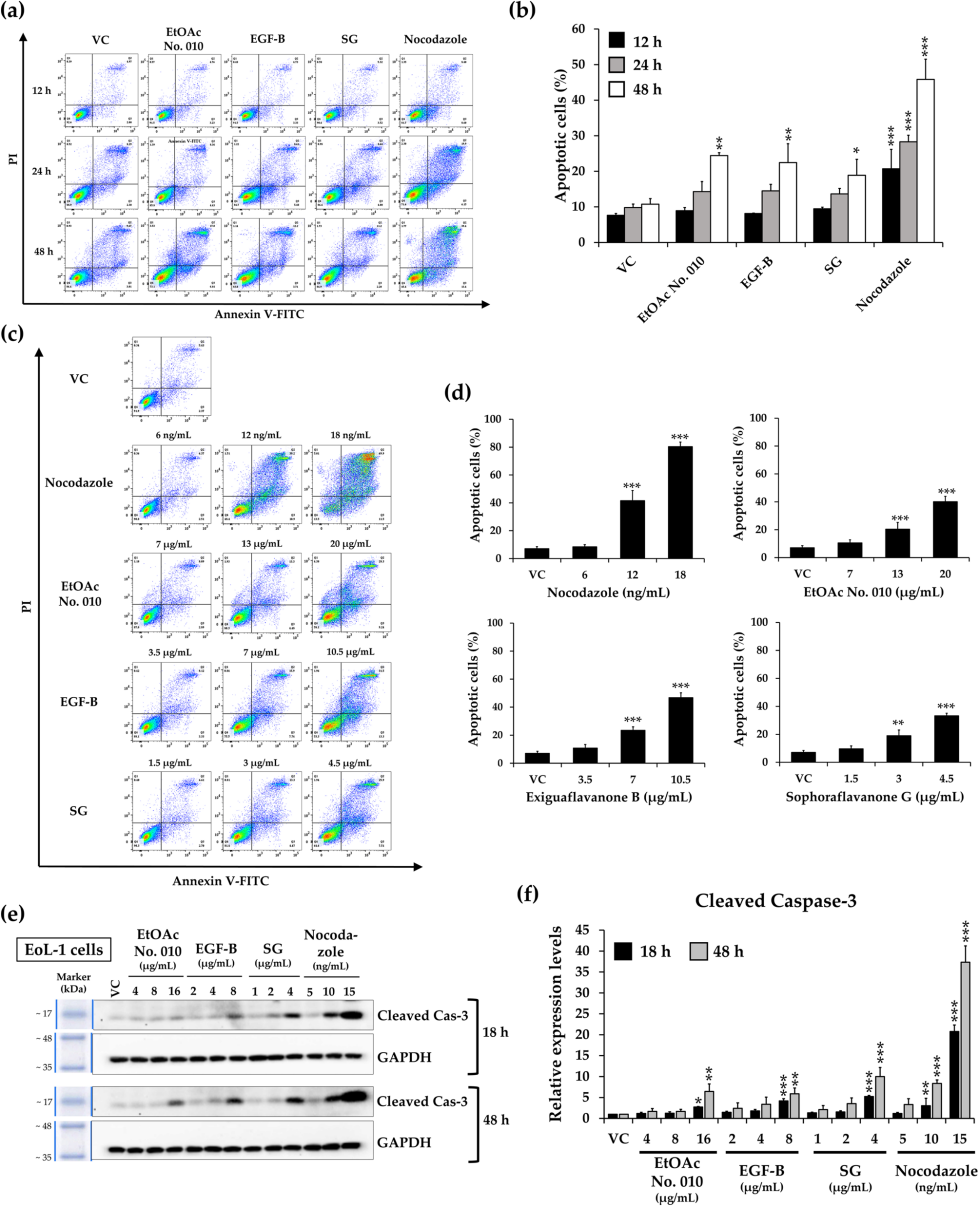
Effect of the crude fractional extract of *S. exigua*, EtOAc No. 010, and the active compounds (EGF-B and SG) on apoptosis induction in EoL-1 cells.** a, b **EoL-1 cells were treated with EtOAc No. 010, EGF-B, SG, and nocodazole (positive control) at their IC_20_ concentrations for 12, 24, and 48 h.** c, d **Additionally, EoL-1 cells were treated with various concentrations of EtOAc No. 010, EGF-B, SG, and nocodazole for 48 h.** e, f **Western blot analysis of cleaved caspase-3 in EoL-1 cells after treatment with the crude fractional extract of *S. exigua* (EtOAc No. 010) and the active compounds (EGF-B and SG) for 18 and 48 h. Band intensity was quantified using a scanning densitometer, with GAPDH as the internal control.** a, c, e **Representative images, and** b, d, f **quantification of independent replicates (n = 3) are shown. Data are expressed as mean ± SD from three independent experiments. *p< 0.05, **p< 0.01, and ***p< 0.001 vs. control group

When cells experience stress, the associated DNA damage induces cell cycle arrest, which may culminate in apoptosis [34]. Due to the accumulation of sub-G1 phase in both cell lines after treatment with the crude fractional extract of *S. exigua* and its active compounds, flow cytometry was used for apoptosis analysis. KG-1a and EoL-1 cells were treated with EtOAc No. 010, EGF-B, SG, and nocodazole (as a positive control) at their IC_50_ concentration for 12, 24, and 48 h, as well as at various concentrations for 48 h. The results showed that EtOAc No. 010, EGF-B, and SG significantly promoted cell apoptosis in a time- and dose-dependent manner (Figs. 6 and 7).

Next, the cleavage of the effector caspase-3 was examined and found to be significantly upregulated in a time- and dose-dependent manner in both KG-1a (Fig. 6e and f) and EoL-1 cells (Fig. 7e and f) after treatment with various concentrations of EtOAc No. 010, EGF-B, and SG for 48 h. These results suggested that EtOAc No. 010 and the active compounds reduced the viability of KG-1a and EoL-1 cells by inducing apoptosis.

### Proteomic analysis of sophoraflavanone G (SG) treated KG-1a leukemic cells

**Fig. 8:**
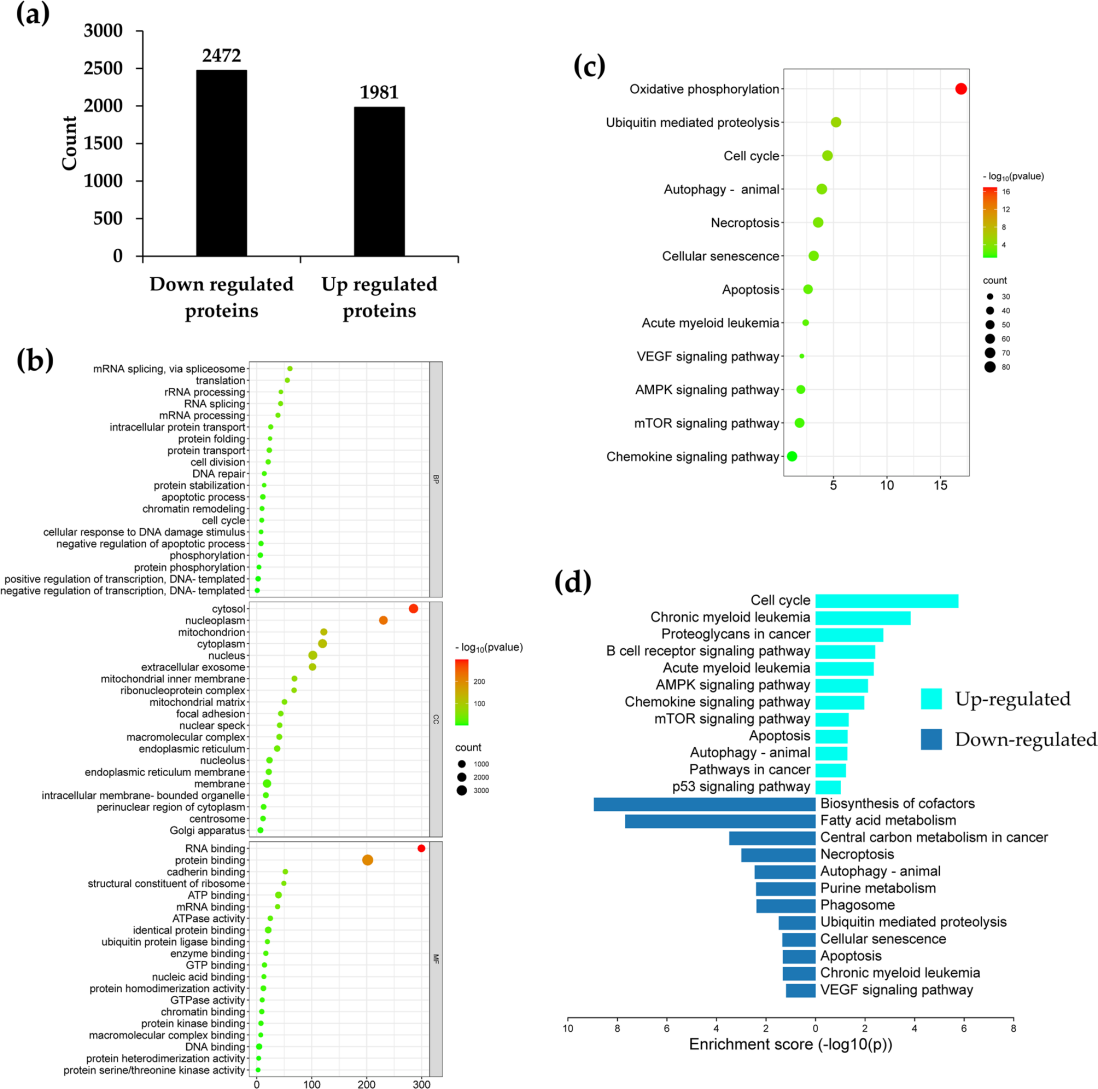
The proteomic analysis of KG-1a cells treated with SG.** a **Histogram showing the number of differentially expressed proteins in KG-1a cells following SG treatment.** b **Top 20 enriched GO terms of differentially expressed proteins, categorized into biological process (BP; upper panel), cellular component (CC; middle panel), and molecular function (MF; lower panel).** c **Bubble plots represent KEGG enrichment analysis of differentially expressed proteins.** d **KEGG pathway enrichment analysis of upregulated proteins and downregulated proteins. Data are from three independent experiments

To investigate the mechanism by which SG induces cell cycle arrest and apoptosis, the KG-1a cell line was used as a leukemic cell model. Cells were treated with IC_20_ concentration of SG and DMSO (vehicle control) for 24 h. Total protein was then extracted and subjected to proteomic analysis. The proteomic findings revealed the identification of 4,455 proteins in both groups. Compared to the control group, SG treatment led to the downregulation of 2,472 proteins and the upregulation of 1,981 proteins (Fig. 8a).

Next, proteomic data were analyzed using bioinformatics and subsequently subjected to gene ontology (GO) enrichment analysis. GO terms are categorized into three groups: biological process (BP), cellular component (CC), and molecular function (MF) [35, 36]. The differentially expressed proteins were enriched in various biological processes, including cell cycle phase and apoptotic processes. GO cellular components analysis revealed significant enrichment of differentially expressed proteins in cytosol, nucleoplasm, mitochondrion, and nucleus. The molecular function terms were enriched in RNA binding and protein binding (Fig. 8b). KEGG pathway analysis showed that the major signal transduction pathways enriched by differentially expressed proteins included the AMPK, mTOR, and VEGF pathways. Additionally, these proteins were also enriched in other KEGG pathways, including oxidative phosphorylation, ubiquitin-mediated proteolysis, cell cycle regulation, autophagy, necroptosis, and apoptosis (Fig. 8c). Furthermore, KEGG analysis of upregulated and downregulated proteins affected by SG revealed that upregulated proteins were associated with the cell cycle, proteoglycans in cancer, the AMPK signaling pathway, and apoptosis. In contrast, downregulated proteins were linked to cofactor biosynthesis, necroptosis, autophagy, and apoptosis (Fig. 8 d).

### Effects of crude fractional extract and active compounds (EGF-B and SG) from *S. exigua* on WT1 expression in KG-1a and EoL-1 leukemic cells

**Fig. 9:**
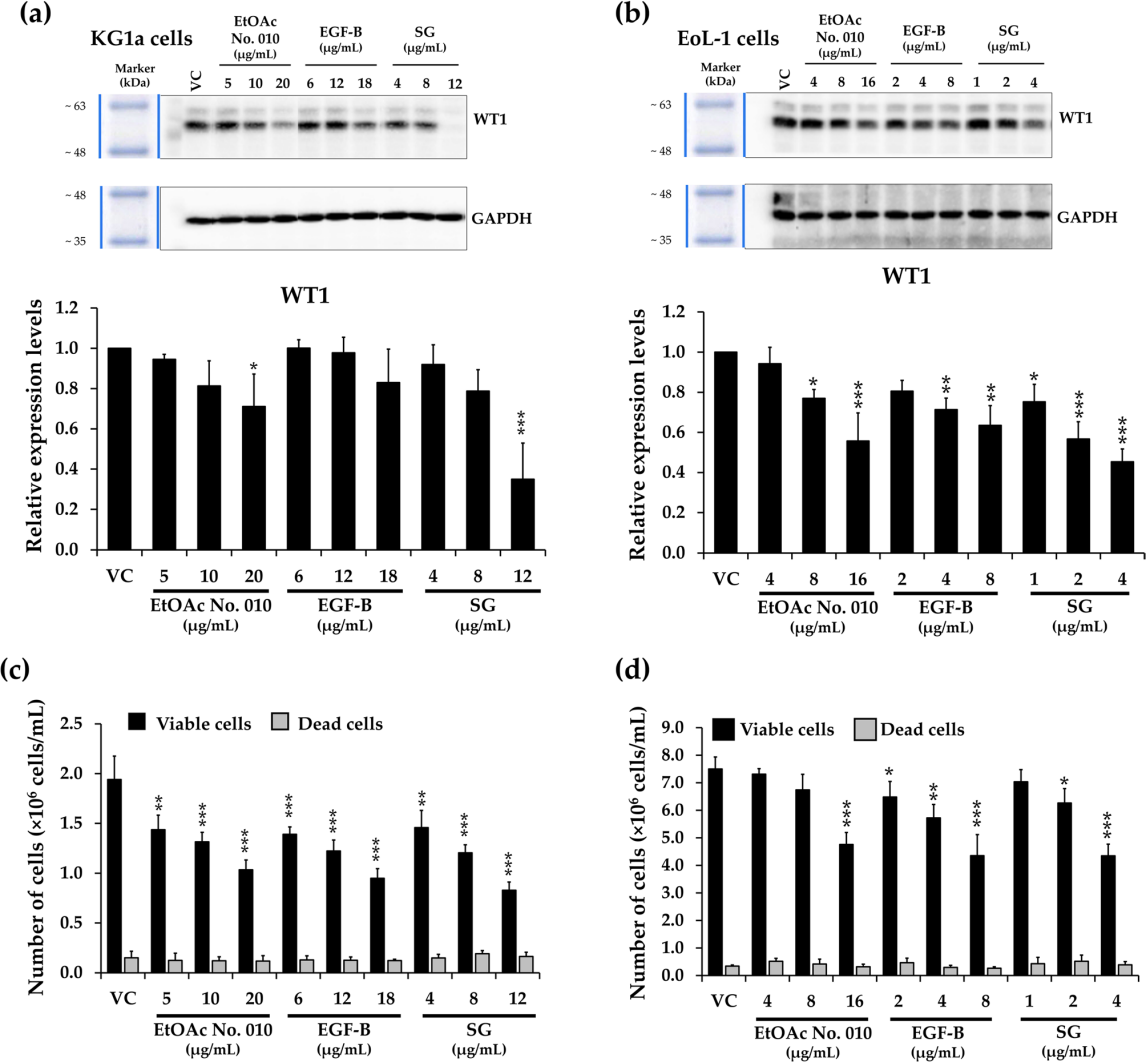
Effects of crude fractional extract and active compounds from *S. exigua* on WT1 expression in KG-1a and EoL-1 cells. WT1 protein expression in **a** KG-1a and **b** EoL-1 cells following treatment with various concentrations of EtOAc No. 010 and active compounds (EGF-B and SG) for 48 h, assessed by Western blotting and analyzed using a scanning densitometer. WT1 levels were normalized to GAPDH protein levels. Total cell number of **c** KG-1a and **d** EoL-1 cells following treatment with the crude fractional extract and active compounds for 48 h, determined using the trypan blue exclusion method. Results expressed as mean ± SD from three independent experiments. Asterisks (*) indicate significant differences compared to the vehicle control (*p < 0.05, **p < 0.01, and ***p < 0.001).

WT1 plays a crucial role in development, differentiation, apoptosis, and proliferation [37]. It is overexpressed in leukemia, promoting cell proliferation and inhibiting apoptosis [38]. The reduction of WT1 expression from high to normal levels is an indicator of complete remission in leukemia patients [39]. To investigate the antiproliferative effect of EtOAc No. 010 and the active compounds (EGF-B and SG) on KG-1a and EoL-1 leukemic cells, both cell Lines were treated with various concentrations of EtOAc No. 010, EGF-B, and SG for 48 h, and WT1 expression was assessed using Western blot analysis. WT1 expression levels were significantly reduced in a dose-dependent manner in both KG-1a and EoL-1 cells following treatment. SG particularly led to a significant decrease in WT1 protein expression, especially in KG-1a cells but also in EoL-1 cells (Fig. 9). WT1 expression in both leukemic cell lines correlated with a reduction in cell number after treatment (Fig. 9c and d). These results suggested that the effects of EtOAc No. 010 and active compounds on WT1 expression may contribute to the repression of leukemic cell proliferation.

## Discussion

Leukemia is characterized by the clonal expansion and the uncontrolled proliferation of leukemic cells in the bone marrow, leading to an excessive numbers of abnormal leukocytes in the peripheral blood [17]. The incidence and mortality rates of leukemia have been increasing each year [18]. Although chemotherapy remains the most effective and widely used treatment, chemotherapeutic drugs also destroy normal cells, leading to numerous side effects. As a result, alternative medicine has gained increasing attention in the treatment of human diseases, including cancer, due to its low toxicity to normal cells. *Sophora exigua* is well known component of Kheaw-Hom, a traditional Thai traditional remedy, used for the treatment of fever, inflammation, and bacterial and viral infections [1, 2]. In this study, *S. exigua* was collected from 12 different locations in Thailand and extracted with Hex, EtOAc, and EtOH. The yields of fractional extracts in this study were observed by less than 20%. According to the previous study, several factors influence plant extract yields [40], particularly those of the *Sophora* genus [41]. Additionally, *Sophora exigua* is an inexpensive raw material. Therefore, although its extract yield is relatively lower than that of other plants, it does not significantly impact production cost. All crude fractional extracts were initially screened for their cytotoxicity against leukemic cell lines and normal cells (PBMCs). Most of the EtOAc crude fractional extracts exhibited anti-proliferative activity against both KG-1a and EoL-1 cells. Among them, EtOAc No. 010 demonstrated strong cytotoxicity in both leukemic cell lines, with IC_50_ values of 45.45 ± 3.25 and 36.33 ± 1.17 µg/mL, respectively, while displaying only mild toxicity towards PBMCs. Additionally, *S. exigua* obtained from different locations contained a diverse array of bioactive compounds, which may have resulted from the variations in natural soil composition and agro-climatic conditions [42, 43]. These factors could contribute to differences in the cytotoxicity of *S. exigua* in leukemic cell lines.

Numerous bioactive compounds have been reported in *S. exigua* root extracts, exhibiting various health-promoting effects, including anti-inflammatory, antimicrobial, antioxidant, and anticancer activities [1, 8]. SG was isolated from EtOAc No. 010 using column chromatography, yielding 10.42%. SG is a well-known compound isolated from the *Sophora* genus and demonstrates multiple biological activities, including antibacterial activity against methicillin-resistant *Staphylococcus aureus* (MRSA), as well as anti-inflammatory, anti-malarial, anti-allergic, and antimicrobial, and anticancer properties. SG, previously isolated from *S. flavescens*, has been identified as a potential small-molecule inhibitor of STAT signaling, the Src family, PI3K/Akt, MAPKs, and NF-κB. It has been found to induce antiproliferative effects and apoptosis in various human cancer cell lines, including human Hodgkin’s lymphoma, multiple myeloma, breast cancer, prostate cancer, epithelial malignant melanoma, lung cancer, and colorectal carcinoma [28]. Furthermore, exiguaflavanones were also isolated from *S. exigua* and have been reported to possess various biological activities. In 2023, Arjsiri et al. identified EGF-B, a significant flavonoid compound, from *S. exigua* roots. The compound exhibited potent anti-cancer properties by inhibiting the invasion of LPS- and ATP-stimulated A549 lung cancer cells via the NLRP3 inflammatory pathway [31]. Similarly, the structure of EGF-B shares the same carbon skeleton orientation as frullanolide, a compound that has demonstrated strong anti-breast cancer activity in the MDA-MB- and MCF-7 breast cancer cell lines [30]. Additionally, EGF-B effectively inhibited the growth of MRSA, completely suppressing all MRSA strains at a concentration of 50 µg/mL [29]. However, the anti-leukemic activities of EGF-B and SG have not yet been fully elucidated. In our study, EGF-B and SG exhibited cytotoxic effects against leukemic cell Lines. Furthermore, we evaluated the inhibitory effect of EtOAc No. 010 and these two active compounds on leukemic cell proliferation. The rates of proliferation demonstrated that they inhibited the growth of KG-1a and EoL-1 cells in a dose- and time-dependent manner. Our findings revealed that EtOAc No. 010, EGF-B, and SG significantly suppressed the proliferation of KG-1a and EoL-1 cells by inducing G1 phase arrest and downregulating the expression of CDK4 (cyclin-dependent kinase), a key G1 phase regulatory protein. These extracts and active compounds also induced apoptosis in both KG-1a and EoL-1 leukemic cells in a time- and dose-dependent manner, accompanied by the upregulation of cleaved caspase-3 expression. Previous studies have reported that SG inhibited the tyrosine phosphorylation of STAT proteins in lymphoma and solid cancer cells, including breast cancer, prostate cancer, epithelial malignant melanoma, lung cancer, and colorectal carcinoma [28]. SG, isolated from *S. flavescens*, induced G0/G1 phase arrest in HL-60 cells following 24 h of treatment [44]. This compound also exhibited cytotoxicity against HL-60 cells by promoting apoptosis through the inhibition of MAPK pathways [45]. Moreover, SG enhanced apoptosis and suppressed cell migration and invasion in triple-negative breast cancer cell lines by targeting the EGFR-PI3K-AKT pathway [46]. Similarly, EGF-B has been reported to induce G1 phase arrest and inhibit A549 proliferation [31].

Additionally, proteomic analysis was performed to identify the differentially expressed genes in KG-1a cells influenced by SG treatment. KEGG pathway analysis revealed that the differentially expressed proteins were enriched in several key pathways, including oxidative phosphorylation, proteolysis, cell cycle, autophagy, and apoptosis. These findings are consistent with the inhibitory effects of SG on leukemic cell proliferation by promoting cell cycle arrest and apoptosis.

WT1 protein plays a crucial role in cancer pathogenesis, including solid tumors and hematological malignancies. Its expression in primary AML blasts is significantly elevated compared with that in normal hematopoietic stem and progenitor cells. This overexpression correlates with poorer clinical outcomes and is associated with leukemia recurrence [23]. WT1 is a key protein involved in promoting cell proliferation. In a study by Zhou et al. (2020), WT1 overexpression was found to enhance the self-renewal capacity of leukemic stem cells (LSCs) and promoted leukemic cell proliferation. Treatment of Kasumi-1 and THP-1 human leukemic cell lines with WP1130 (a deubiquitinase inhibitor) demonstrated potential anti-leukemic activity by inducing apoptosis and inhibiting the self-renewal of LSCs through the suppression of BCL2L2 (B cell lymphoma 2 family) [38]. In contrast to previous studies, several natural compounds, including curcumin, a natural flavonoid from *Curcuma longa* Linn., have been shown to inhibit leukemic cell proliferation by downregulating WT1 expression via the PKC-α signaling pathway [47]. In this study, the crude fractional extract and active compounds from *S. exigua* were investigated for their inhibitory effect on leukemic cell proliferation by downregulating WT1 protein expression. EtOAc No. 010 and the two active compounds significantly suppressed WT1 protein expression in both cell lines in a dose-dependent manner. This suppression was associated with a reduced number of leukemic cells. Among them, SG exhibited the strongest inhibition of WT1 protein expression in both KG-1a cells and EoL-1 cells. These findings are consistent with previous results regarding the altered rate of proliferation. Similarly, KEGG enrichment analysis revealed that the primary signaling pathway enriched were the AMPK, VEGF, and mTOR pathways. The AMPK pathway not only plays a crucial role in cellular metabolism but also regulates cancer progression by inhibiting cell proliferation, migration, and invasion, while promoting apoptosis [48]. In 2021, Meng et al. investigated the specific mechanisms induced by WT1 in ovarian cancer using transcriptome technology and bioinformatics analysis [49]. The AMPK pathway was identified as one of the key signal transduction pathways and exhibited a complex interaction with WT1 protein. Previous studies, AMPK/mTOR pathway plays a role in autophagy activation in hepatocellular carcinoma following treatment with glycochenodeoxycholate [50]. Additionally, the AMPK pathway has been found to induce apoptosis in ovarian cancer cells and regulate cell migration and invasion [51]. Moreover, the VEGF pathway plays a crucial role in angiogenesis and is overexpressed in certain solid cancers. In 75% of AML patients, WT1 was co-expressed with VEGF. A reduction in VEGF expression during chemotherapy was associated with a decrease in WT1 levels [52]. Therefore, these pathways may be linked to WT1 protein expression.

Previous studies have reported that individual variability among patients and intra-tumoral heterogeneity contribute to differences in clinical outcomes and treatment efficacy in cancer therapy. Likewise, phenotypic diversity among cell lines leads to varying responses to drug treatment [53, 54]. Therefore, the effects of SG and EGF-B are particularly important and beneficial for leukemia treatment, especially in suppressing WT1 protein expression.

## Conclusion

In conclusion, this study investigated the effects of *Sophora exigua* crude fractional extracts and its active compounds on leukemic cells, KG-1a and EoL-1 cells. Among 36 different crude fractional extracts, EtOAc No. 010 was the most effective, exhibiting cytotoxicity against both KG-1a and EoL-1 cells. These effects were attributed to the active compounds SG and EGF-B. EtOAc No. 010, SG, and EGF-B demonstrated strong anti-leukemic activity by arresting cell cycle at the G1 phase and inducing cell apoptosis. Furthermore, they suppressed the expression of WT1 protein, contributing to the inhibition of leukemic cell proliferation. However, SG exhibited greater activity than EGF-B. These findings suggest that SG could serve as a therapeutic agent for the prevention of leukemogenesis.

## Supplementary information

Supplementary Material 1. Supplement figure 1. (a) HMBC, (b) HMQC, and (c) HSQC NMR spectrums of SG.

Supplementary Material 2

## Data Availability

The datasets used and/or analyzed during the current study are available from the corresponding author by reasonable request.
