# Supplementary material for: Sophoraflavanone G from Phit-Sanat (Sophora Exigua Craib) inhibits WT1 protein expression and induces cell cycle arrest and apoptosis in acute myeloid leukemia
Source: BMC Complement Med Ther. 2025 Oct 8;25:362. doi: 10.1186/s12906-025-05116-1 (PMC12506424; doi:10.1186/s12906-025-05116-1)
Supplement: Supplementary file 1 — Supplementary Material 1. Supplement figure 1. (a) HMBC, (b) HMQC, and (c) HSQC NMR spectrums of SG. [file 12906_2025_5116_MOESM1_ESM.pptx]

## Slide 1
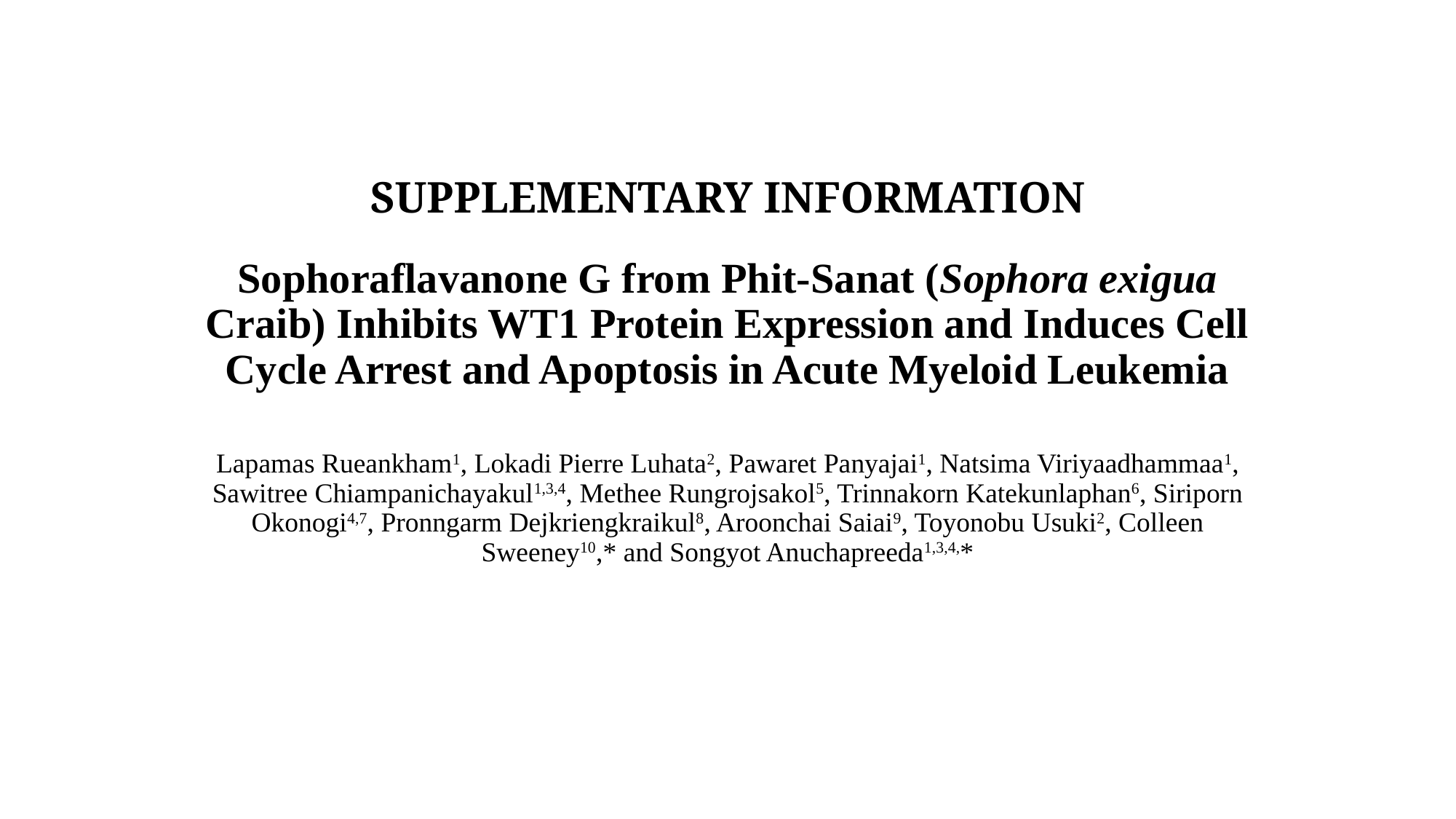

SUPPLEMENTARY INFORMATION
# Sophoraflavanone G from Phit-Sanat (Sophora exigua Craib) Inhibits WT1 Protein Expression and Induces Cell Cycle Arrest and Apoptosis in Acute Myeloid Leukemia
Lapamas Rueankham1, Lokadi Pierre Luhata2, Pawaret Panyajai1, Natsima Viriyaadhammaa1, Sawitree Chiampanichayakul1,3,4, Methee Rungrojsakol5, Trinnakorn Katekunlaphan6, Siriporn Okonogi4,7, Pronngarm Dejkriengkraikul8, Aroonchai Saiai9, Toyonobu Usuki2, Colleen Sweeney10,* and Songyot Anuchapreeda1,3,4,*

## Slide 2
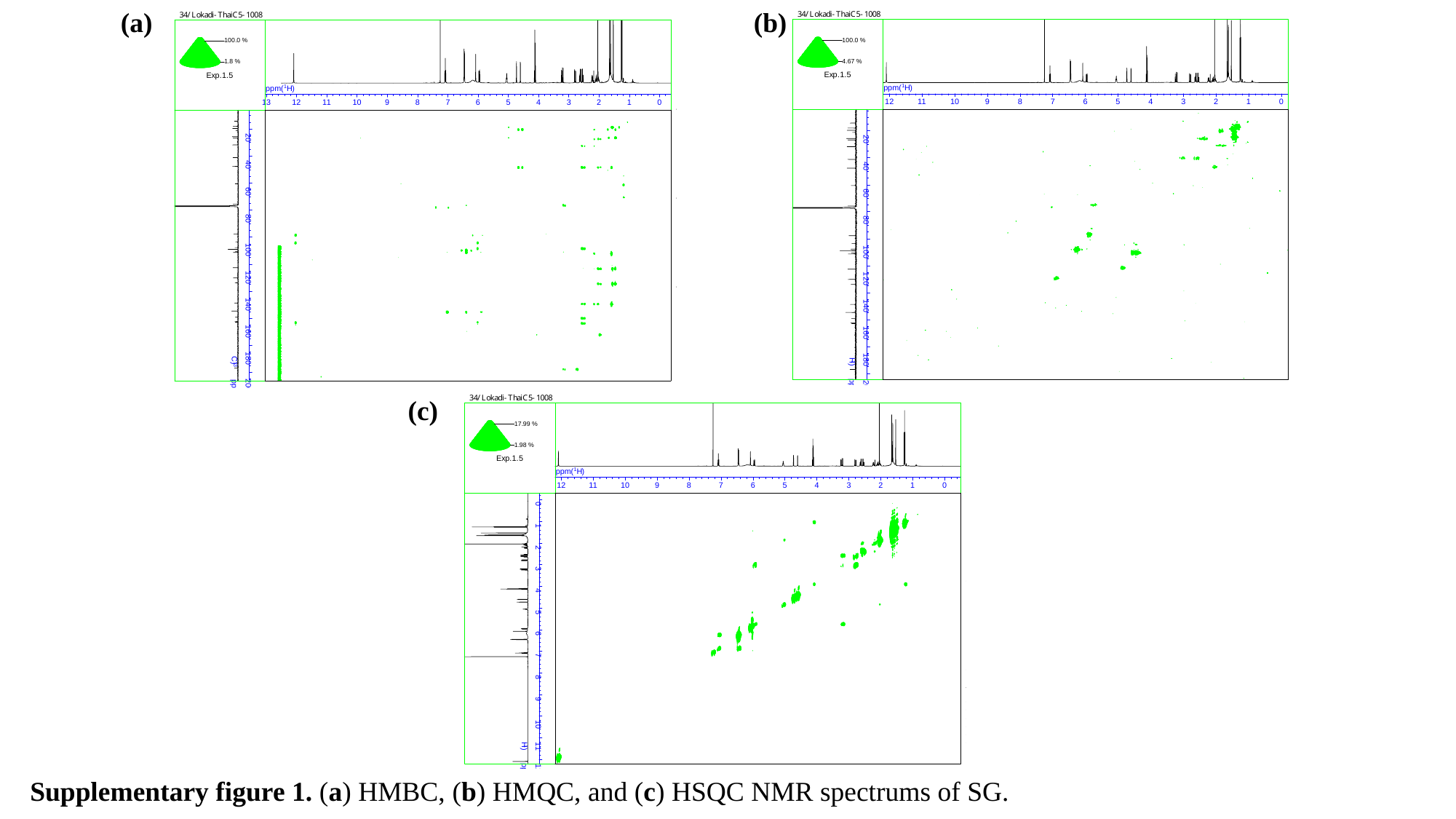

(a)
(b)
(c)
Supplementary figure 1. (a) HMBC, (b) HMQC, and (c) HSQC NMR spectrums of SG.
