# Supplementary material for: Sophoraflavanone G from Phit-Sanat (Sophora Exigua Craib) inhibits WT1 protein expression and induces cell cycle arrest and apoptosis in acute myeloid leukemia
Source: BMC Complement Med Ther. 2025 Oct 8;25:362. doi: 10.1186/s12906-025-05116-1 (PMC12506424; doi:10.1186/s12906-025-05116-1)
Supplement: Supplementary file 2 — Supplementary Material 2 [file 12906_2025_5116_MOESM2_ESM.pdf]

KG-1a cells

WT1

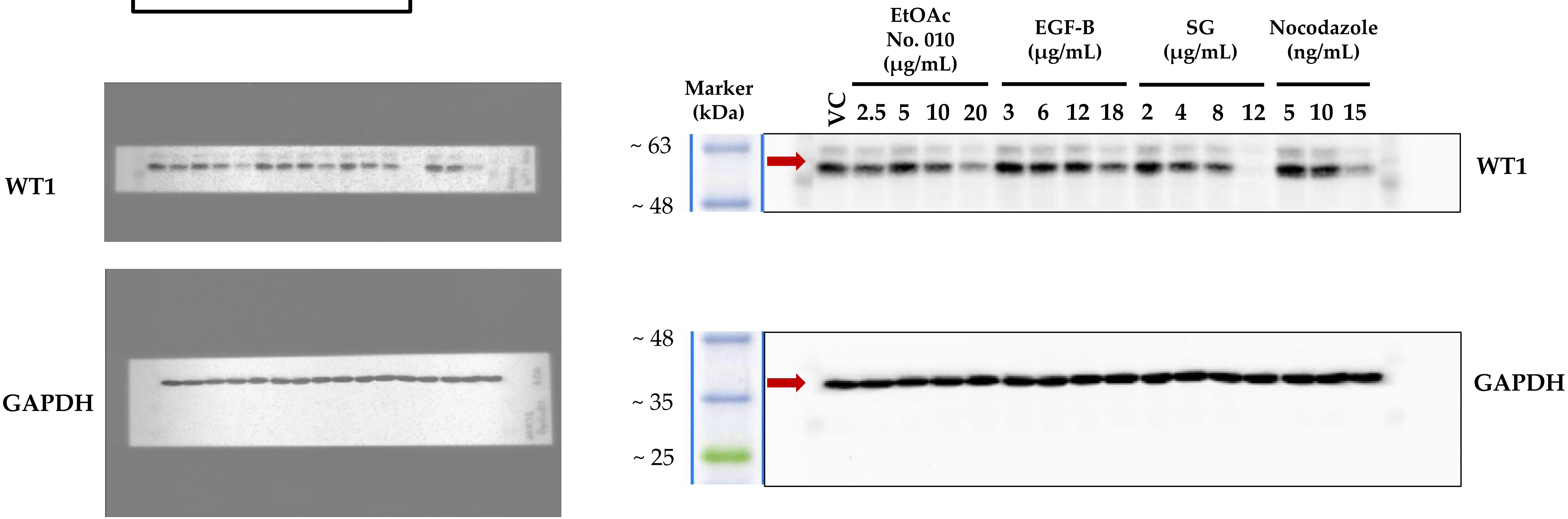

KG-1a cells

24 h

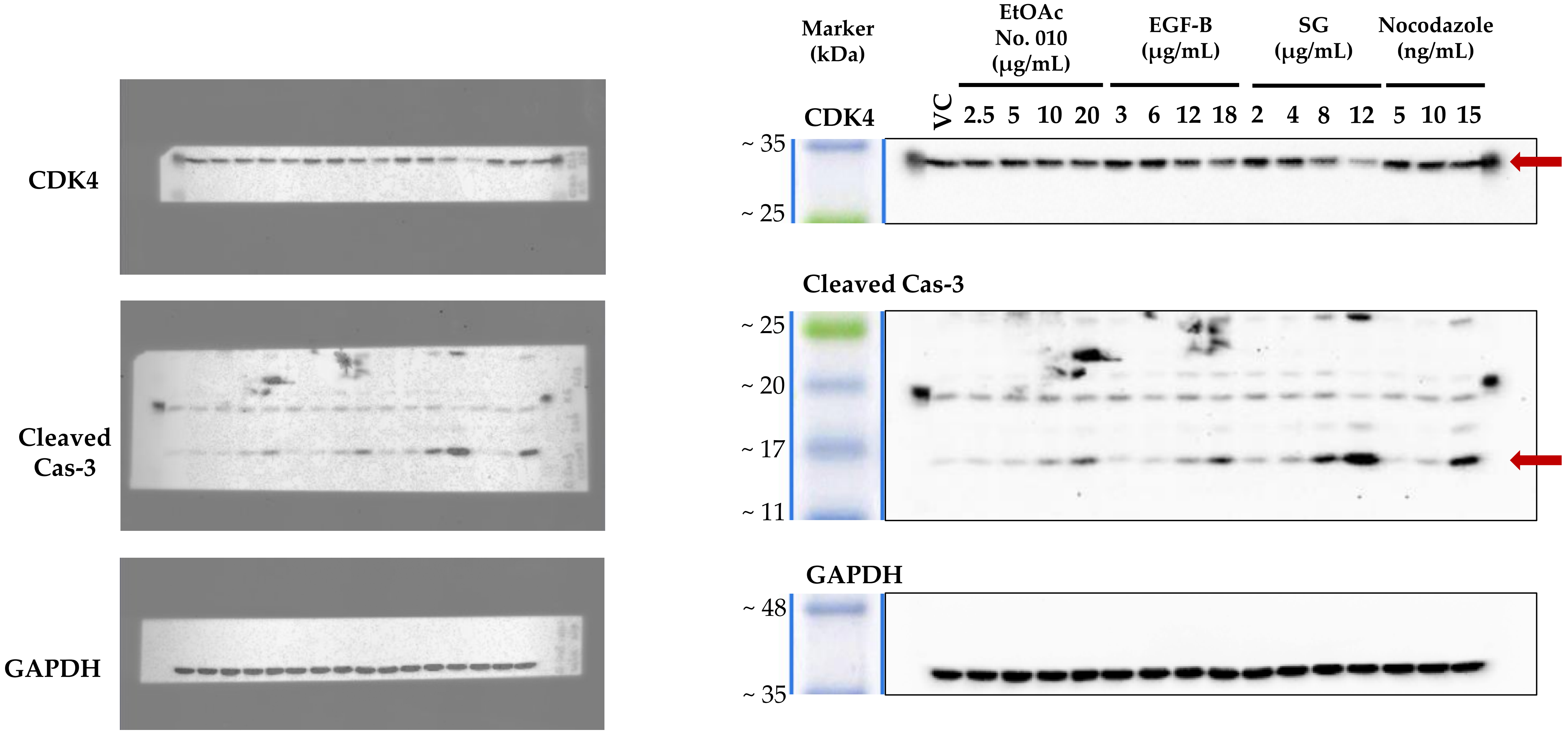

KG-1a cells

48 h

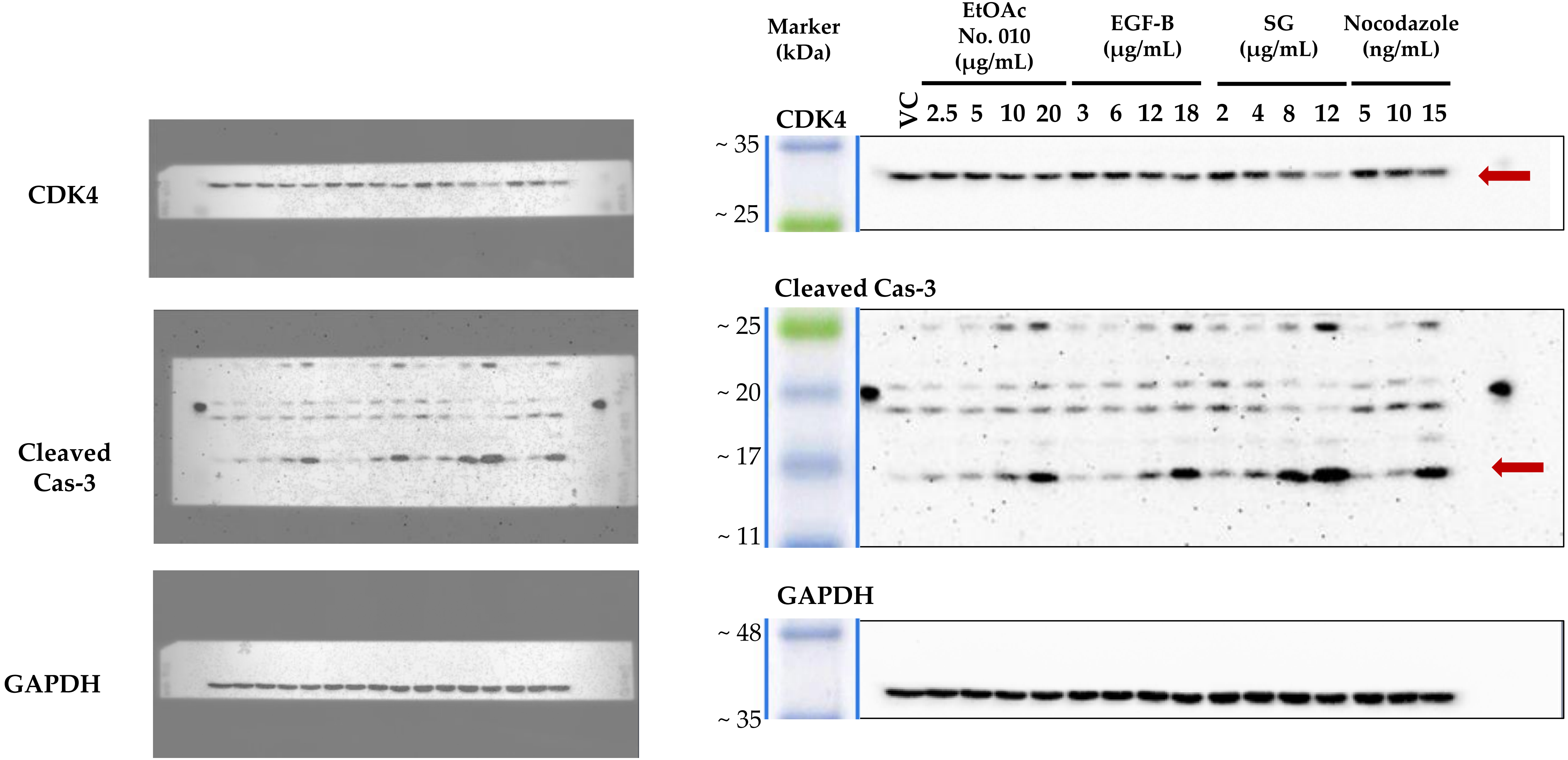

EoL-1 cells

WT1

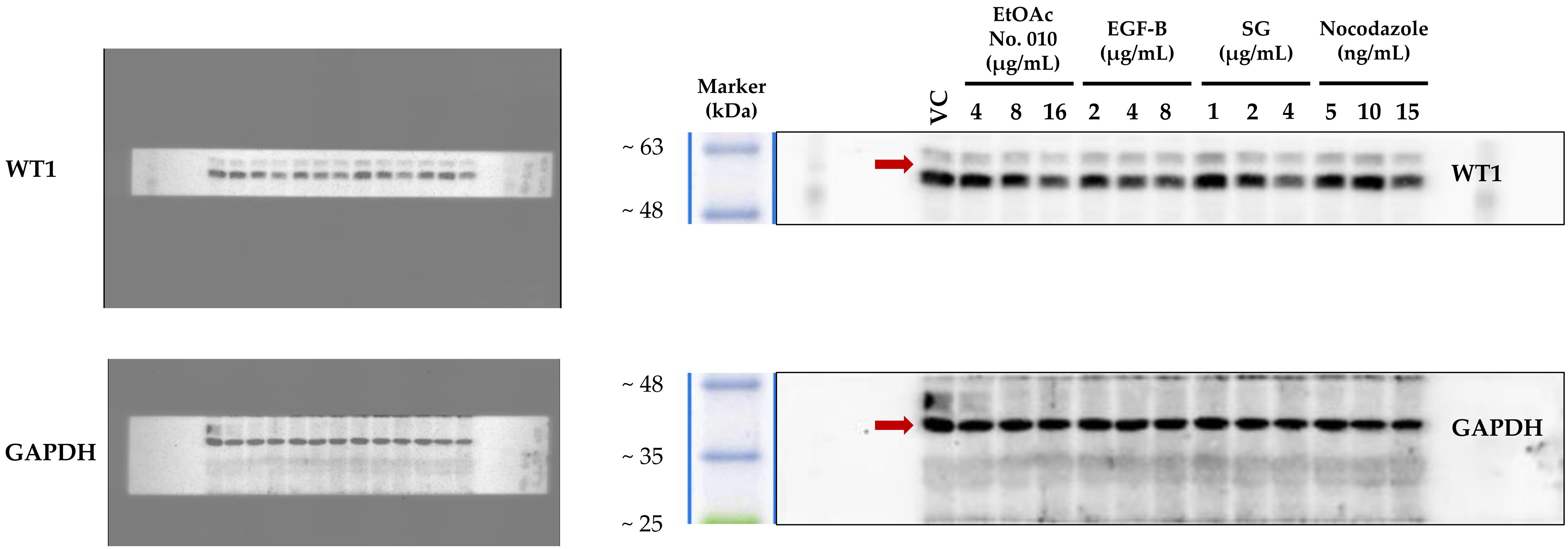

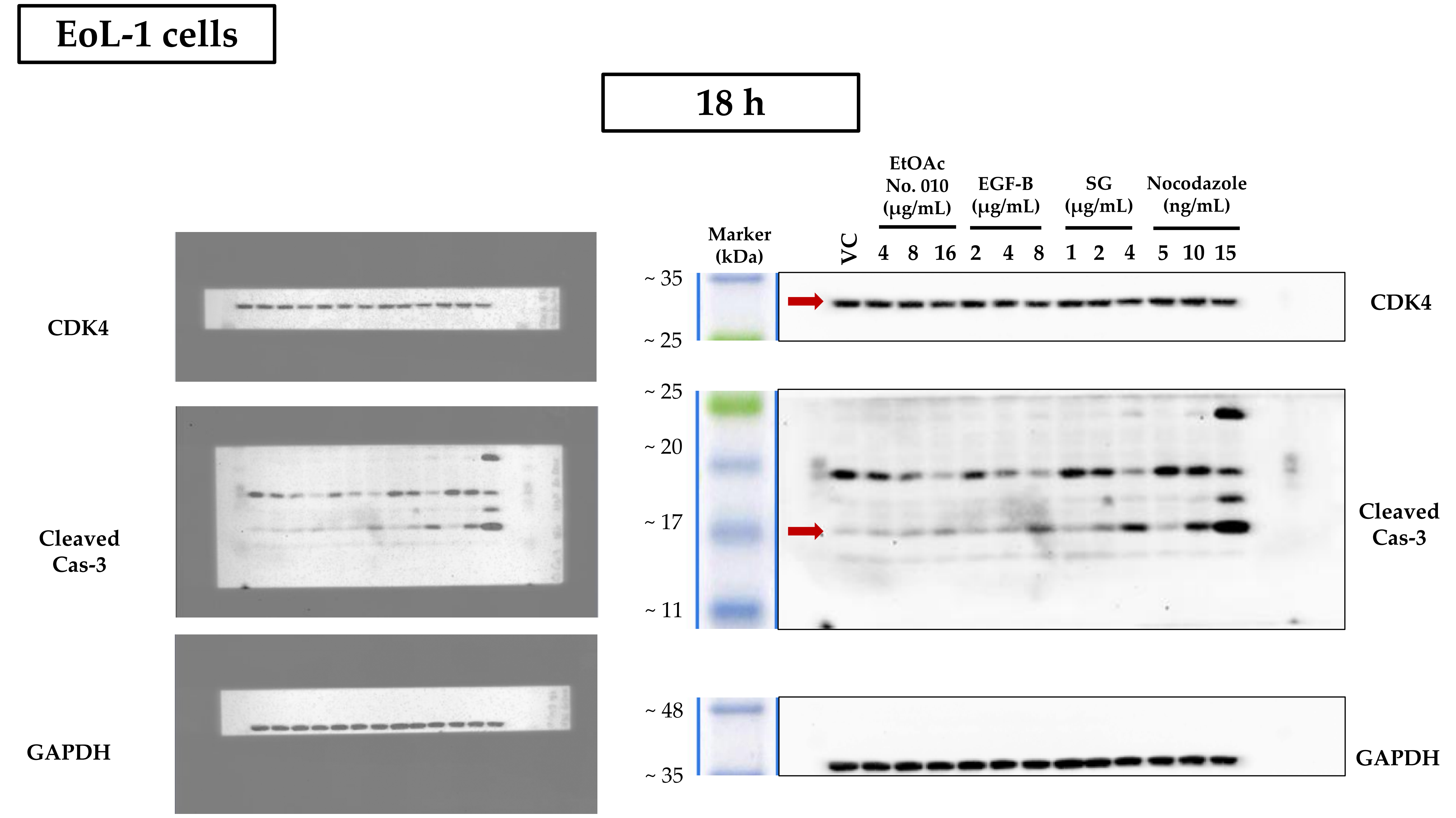

EoL-1 cells
18 h
EtOAc
No. 010
(μg/mL)
EGF-B
(μg/mL)
SG
(μg/mL)
Nocodazole
(ng/mL)
Marker
(kDa)
VC
4 8 16 2 4 8 1 2 4 5 10 15
~ 35
~ 25
~ 25
~ 20
~ 17
~ 11
~ 48
~ 35
CDK4
Cleaved
Cas-3
GAPDH
CDK4
Cleaved
Cas-3
GAPDH

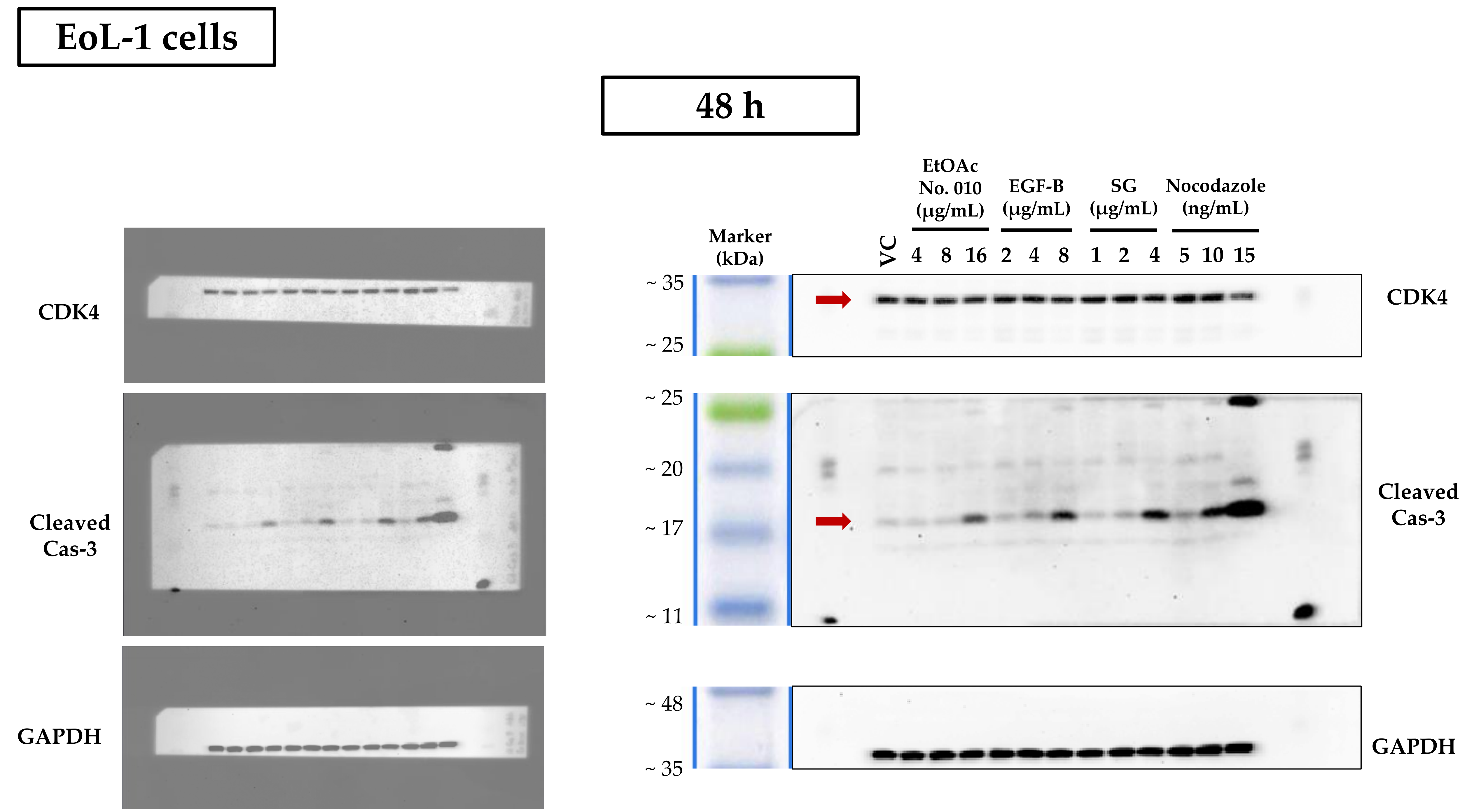
EoL-1 cells
48 h
CDK4
Cleaved Cas-3
GAPDH
Marker (kDa)
VC
EtOAc No. 010 (μg/mL)
4 8 16
EGF-B (μg/mL)
2 4 8
SG (μg/mL)
1 2 4
Nocodazole (ng/mL)
5 10 15
~ 35
~ 25
~ 25
~ 20
~ 17
~ 11
~ 48
~ 35
CDK4
Cleaved Cas-3
GAPDH
